# Quality‐control of an hourly rainfall dataset and climatology of extremes for the UK

**DOI:** 10.1002/joc.4735

**Published:** 2016-04-24

**Authors:** Stephen Blenkinsop, Elizabeth Lewis, Steven C. Chan, Hayley J. Fowler

**Affiliations:** ^1^ Water Resource Systems Research Laboratory, School of Civil Engineering and GeosciencesNewcastle UniversityNewcastle upon TyneUK; ^2^ Hadley CentreMet OfficeExeterUK

**Keywords:** intense rainfall, extremes, UK, flash floods, hourly rainfall, quality control

## Abstract

Sub‐daily rainfall extremes may be associated with flash flooding, particularly in urban areas but, compared with extremes on daily timescales, have been relatively little studied in many regions. This paper describes a new, hourly rainfall dataset for the UK based on ∼1600 rain gauges from three different data sources. This includes tipping bucket rain gauge data from the UK Environment Agency (EA), which has been collected for operational purposes, principally flood forecasting. Significant problems in the use of such data for the analysis of extreme events include the recording of accumulated totals, high frequency bucket tips, rain gauge recording errors and the non‐operation of gauges. Given the prospect of an intensification of short‐duration rainfall in a warming climate, the identification of such errors is essential if sub‐daily datasets are to be used to better understand extreme events. We therefore first describe a series of procedures developed to quality control this new dataset. We then analyse ∼380 gauges with near‐complete hourly records for 1992–2011 and map the seasonal climatology of intense rainfall based on UK hourly extremes using annual maxima, n‐largest events and fixed threshold approaches. We find that the highest frequencies and intensities of hourly extreme rainfall occur during summer when the usual orographically defined pattern of extreme rainfall is replaced by a weaker, north–south pattern. A strong diurnal cycle in hourly extremes, peaking in late afternoon to early evening, is also identified in summer and, for some areas, in spring. This likely reflects the different mechanisms that generate sub‐daily rainfall, with convection dominating during summer. The resulting quality‐controlled hourly rainfall dataset will provide considerable value in several contexts, including the development of standard, globally applicable quality‐control procedures for sub‐daily data, the validation of the new generation of very high‐resolution climate models and improved understanding of the drivers of extreme rainfall.

## 1 Introduction

One of the most significant potential consequences of climate change in many parts of the world is the increased occurrence of flooding, which may be associated with prolonged periods of above average rainfall or with an intensification of rainfall extremes (Trenberth *et al.*, 2003). In the UK significant impacts have been associated with flash flooding due to short, intense periods of rainfall (Archer and Fowler, 2015). This may affect rural areas in rapid response catchments; for example, in August 2004, intense rainfall of ∼50 mm h^−1^ at Boscastle in SW England resulted in over 100 m^3^ s^−1^ of flood water and a 3–4.5 m surge, with homes flooded, cars swept away and ∼100 people needing assistance to safety (Doe, 2004). Urban areas are also vulnerable; for example, ∼50 mm of rain fell in around 2 h over Newcastle upon Tyne in northern England on 28 June 2012 (Smith *et al.*, 2015), resulting in over 1200 properties being affected by flooding and up to £8 m in damages to highways (Newcastle City Council, 2013). The UK Climate Change Risk Assessment (Defra, 2012) has estimated that the combined domestic and commercial insurance claims for flood‐related damage may increase almost threefold by the 2050s (central estimate, medium emissions scenario).

The intensification of rainfall with a warming atmosphere arises due to the capacity of warmer air to hold more water than cooler air and therefore to potentially provide more moisture to rainfall events. The Clausius–Clapeyron (CC) relation states that if relative humidity remains constant, then atmospheric humidity will increase at a rate that follows the saturation vapour pressure dependency on temperature – a rate of ∼6–7% °C^−1^ of surface warming (e.g. Allen and Ingram, 2002; Pall *et al.*, 2007). Observational evidence suggests that the intensities of sub‐daily extreme rainfall are more sensitive to changes in temperature compared with those on a daily timescale, with a scaling of rainfall intensity with temperature exceeding that of CC, so called super CC scaling (e.g. Lenderink and van Meijgaard, 2008; Hardwick Jones *et al.*, 2010). However, Blenkinsop *et al.* (2015) have shown a scaling approximating CC for hourly rainfall extremes and temperature in summer for the UK, although this has some dependency on the weather type. A review of empirical studies of the relationship between temperature and short‐duration extreme rainfall is provided in Westra *et al.* (2014).

It is possible that changes and variability in daily extremes may not reflect those at sub‐daily durations (Jakob *et al.*, 2011a; Westra *et al.*, 2014), and so, given the potential for an intensification of short‐duration rainfall, a detailed understanding of current sub‐daily climatologies and their drivers is a prerequisite for any assessment of change in future extreme rainfall and associated hazards. This is evidently dependent on the provision of high‐quality observations, something lacking for many regions of the world (Westra *et al.*, 2014), and so an understanding of recent changes and the important processes and drivers has been limited when compared with daily timescales. Some regional studies have been undertaken, for example, for India (Sen Roy, 2009; Deshpande *et al.*, 2012), South Africa (Sen Roy and Rouault, 2013), Australia (Jakob *et al.*, 2011b; Westra and Sisson, 2011), Japan (Fujibe *et al.*, 2005), Hong Kong (Lenderink *et al.*, 2011), the United States (Muschinski and Katz, 2013), The Netherlands (Lenderink *et al.*, 2011) and Ireland (Leahy and Kiely, 2011). Generally, the examination of sub‐daily precipitation has focussed on the scaling relationship of rainfall intensities with temperature (e.g. Lenderink and van Meijgaard, 2008; Hardwick Jones *et al.*, 2010; Lenderink *et al.*, 2011; Berg *et al.*, 2013). The HadISD dataset (Dunn *et al.*, 2012) also provides global coverage of sub‐daily data for a number of climate variables but, in the case of precipitation, has not been subjected to any quality‐control procedures.

For the UK, there has been considerable research into the climatology of daily precipitation but relatively little on sub‐daily timescales. Jenkins *et al.* (2008) provide long‐term averages of mean precipitation and rain day frequency (daily rainfall ≥ 1 mm) along with 5 km gridded monthly datasets of these variables and of the frequency of days where rainfall ≥10 mm, derived from the Met Office archive of UK daily rainfall observations (maps of these variables may be accessed at the UK Met Office http://www.metoffice.gov.uk/public/weather/climate/). Maraun *et al.* (2009) used 1‐day annual maxima (AM) to demonstrate a predominant east–west pattern to 10‐ and 100‐year return levels, with higher values along the west coast, the western Highlands and the Lake District and with the lowest values in the southeast. However, sub‐daily rainfall data is predominantly gathered from operational networks of rain gauges for flash flood forecasting and has not been used extensively for climatological analysis. Faulkner (1999) provides some information on sub‐daily rainfall extremes but only considers AM, producing UK‐wide estimates of *R*_med_ (median of annual maximum rainfalls) for eight durations between 1 h and 8 days (see also Faulkner and Prudhomme, 1998) from which design rainfall estimates are derived for a range of return periods (RPs). This is, however, based on gauge record lengths as short as 9 years, and so, consequently, the examination of historical changes in UK precipitation extremes has been limited to analyses at daily and longer timescales. Such research identifies more intense winter rainfall since the 1960s (Osborn *et al.*, 2000; Osborn and Hulme, 2002; Maraun *et al.*, 2008), with some evidence of a longer‐term trend (Osborn *et al.*, 2000; Simpson and Jones, 2014). The contribution to winter rainfall from heavy precipitation events has also increased (Jenkins *et al.*, 2008). In contrast, summer daily rainfall events have shown little change or decline in intensity, whilst longer duration events (multi‐day) have increased (Fowler and Kilsby, 2003a, 2003b; Jones *et al.*, 2013).

In this paper, we describe three sources of sub‐daily rain gauge data for the UK, which have been collated and subjected to a series of quality‐control (QC) procedures. These procedures identify potentially suspect values, particularly extremes, based on identified problems associated with sub‐daily data. The combined, quality‐controlled dataset is then used to provide a climatology of the main features of hourly extremes for the period 1992–2011. This climatology describes the frequency, intensity, seasonality and diurnal cycle of extreme hourly rainfall events using AM, *n*‐largest and fixed threshold approaches.

## 2 Data, QC and methodology

### 2.1 Data sources

**Figure 1:**
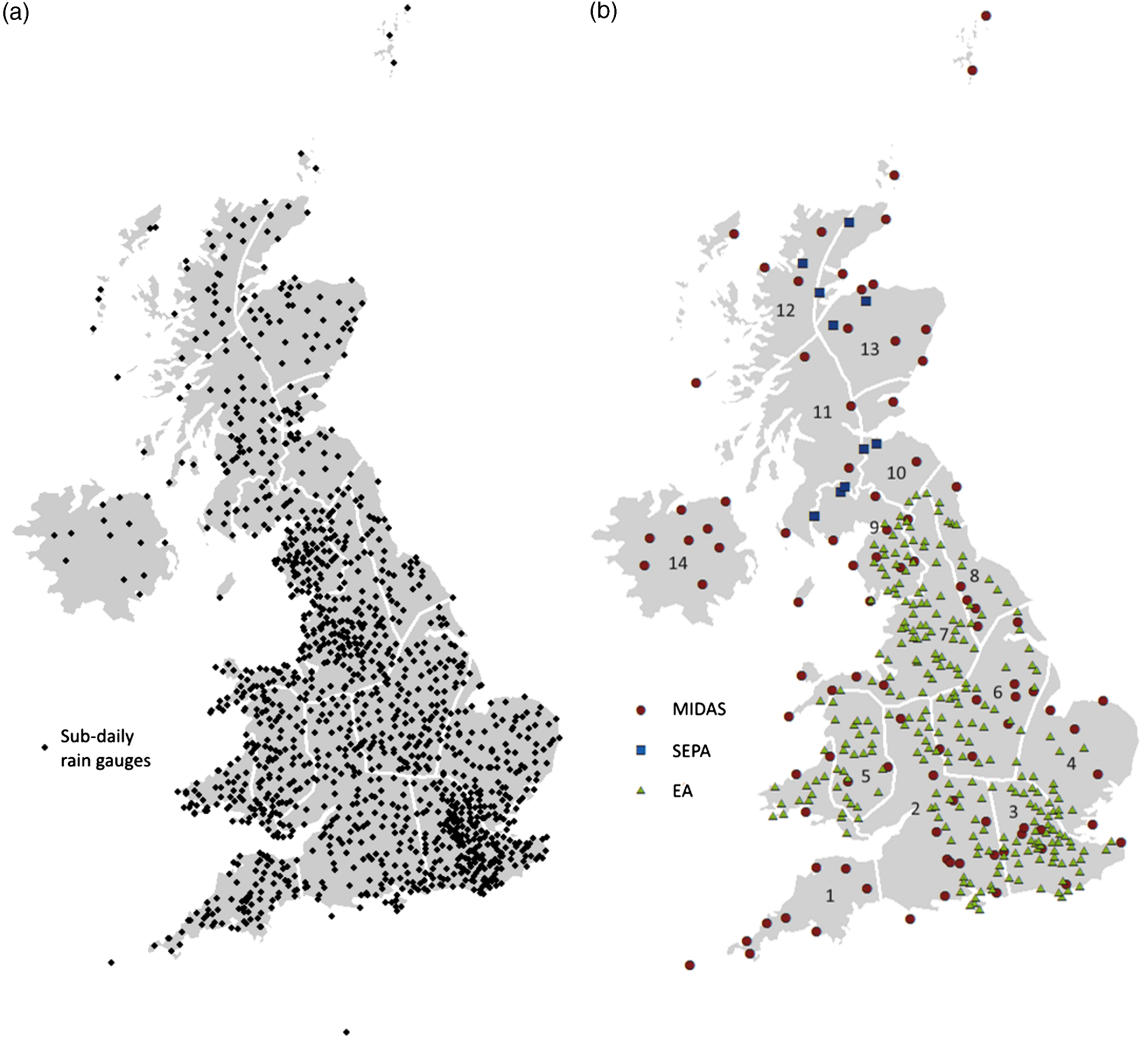
Distribution of hourly rain gauge data (a) before QC procedures and data length requirements were applied and (b) after QC procedures and meeting completeness criteria for the period 1992–2011 as detailed in the main text. The symbols denote the data source, and not all stations are used for each season. The regions shown are the extreme rainfall regions defined by Jones et al. (2014) named: (1) South West, (2) West Country, (3) South East, (4) East Anglia, (5) Mid Wales, (6) Humber, (7) North West, (8) North East, (9) Solway, (10) Forth, (11) South Scotland, (12) North Highland and Islands, (13) East Scotland, (14) North Ireland.

The historical dataset used in this analysis has been constructed from three sources. The first of these is the UK Met Office Integrated Data Archive System (MIDAS), which may be downloaded from the British Atmospheric Data Centre (Met Office, 2012). This data comprises land surface and marine surface observations from the UK Met Office station network and other worldwide stations. In this instance, hourly (1 h) rainfall accumulations from UK locations with records spanning a minimum of 10 years were extracted (216 in total), although additional gauges of shorter duration are available. This limitation was applied as a pragmatic balance between download and processing time and the provision of useable data. A second dataset of hourly rainfall accumulations for 141 gauges was obtained from the Scottish Environmental Protection Agency (SEPA) and the third, comprising tipping bucket rain gauge (TBR) data from ∼1300 gauges across England and Wales, was obtained from the UK Environment Agency (EA). These are largely deployed for operational purposes and have not been previously examined for climatological analyses, although AM time series – primarily from the Midlands region – were used to provide rainfall frequency estimates in the Flood Estimation Handbook (FEH; Faulkner, 1999). The locations of the rain gauges from all sources used here are shown in Figure 1(a).

### 2.2 Pre‐existing data QC

The MIDAS dataset has been subjected to basic QC at each observing site to ensure that some errors are trapped before being transmitted. Data range and self‐consistency checks are performed to ensure that the meteorological values do not lie outside long‐term climatological extremes (Met Office, 2012) and metadata flags are provided for identified suspicious and accumulated values. Although no QC information was available for the SEPA data, the EA TBR data has been subjected to some initial, internal QC procedures using check gauges against which TBR totals *may* be compared (C. Langley, 2013; personal communication). Where the TBR totals are less than (greater than) 25 mm and are within ±2 mm (±8%) of the check gauge, then data are classified as ‘good’. Where differences exceed these tolerances, then data are either classified as ‘suspect’ or, in serious cases, are deleted and marked as missing. Data is also marked suspect in instances where a site inspection has indicated some irregularity with the rain gauge. The results of these procedures are provided as metadata and, in most cases (on average ∼62.5% of all recorded rainfall amounts per rain gauge) are identified as good, although it was noted that this flag is applied to validated daily or monthly accumulations. In this analysis, all data classified as suspect (∼9.5% of rainfall amounts) were treated as missing, whilst all good and ‘unchecked’ (∼28% of rainfall amounts) data were subjected to the additional QC procedures described below. Most of the suspect amounts (∼92%) are 0.2 mm or less (Table 1), which reflects the predominance of 0.2 mm TBRs in use by the EA, but these QC procedures also identify some suspect 15‐min totals and longer accumulations. However, it is evident that a number of unrealistic totals are not denoted as suspect (e.g., >300 recorded amounts of >1000 mm were identified, which could be attributable to a number of possible causes), indicating a need for additional QC procedures.

**Table 1:** Frequency (n) of recorded rainfall amounts (R) identified as suspect in EA QC metadata.

*R* (mm)	≤0.2	≤0.5	≤1	≤2	≤5	≤10
*n*	48 50 529	216 079	164 810	35 754	11 492	2586
*R* (mm)	≤20	≤50	≤100	≤150	>150	
*n*	573	103	7	103	2	

The lower bounds of each class are determined by the upper bound of the preceding class (apart from extreme classes).

**Figure 2:**
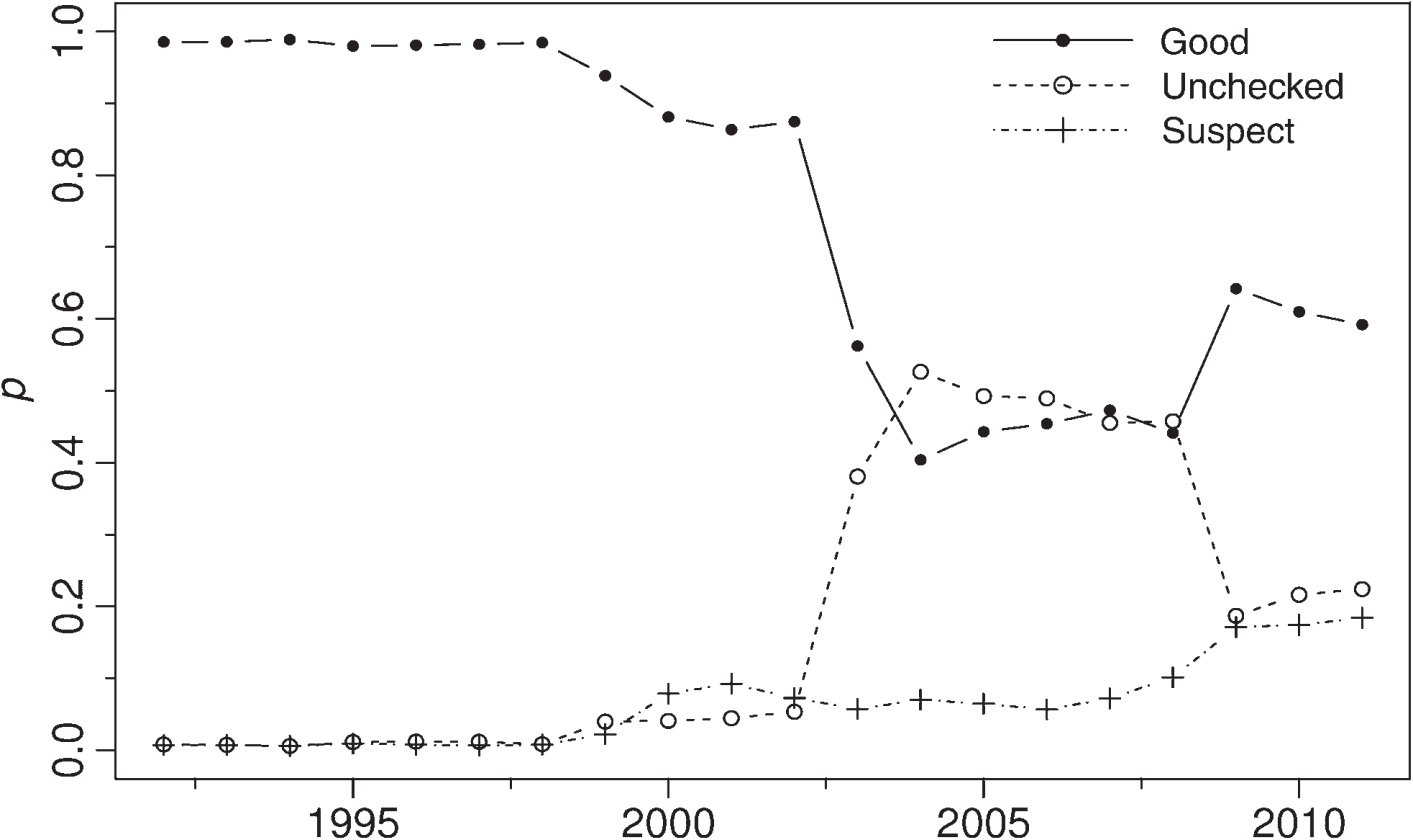
Time series of the mean proportion (p) of the three classes of QC metadata for EA gauges.

A more detailed examination of the EA rain gauge metadata (Figure 2) indicates that its attributes change over time with two very clear patterns:

- A sharp decrease in the proportion of data assessed as good over the period 2003–2008, largely as a consequence of an increase in the proportion of unchecked data followed by only a modest recovery in the proportion of good data;
- a gradual, moderate increase in data identified as suspect.

Further analysis of this metadata presented in Figure S1 (Supporting Information) also suggests regional differences in recording and/or validation procedures and confirms the need for additional, consistent QC.

### 2.3 Additional QC procedures

**Figure 3:**
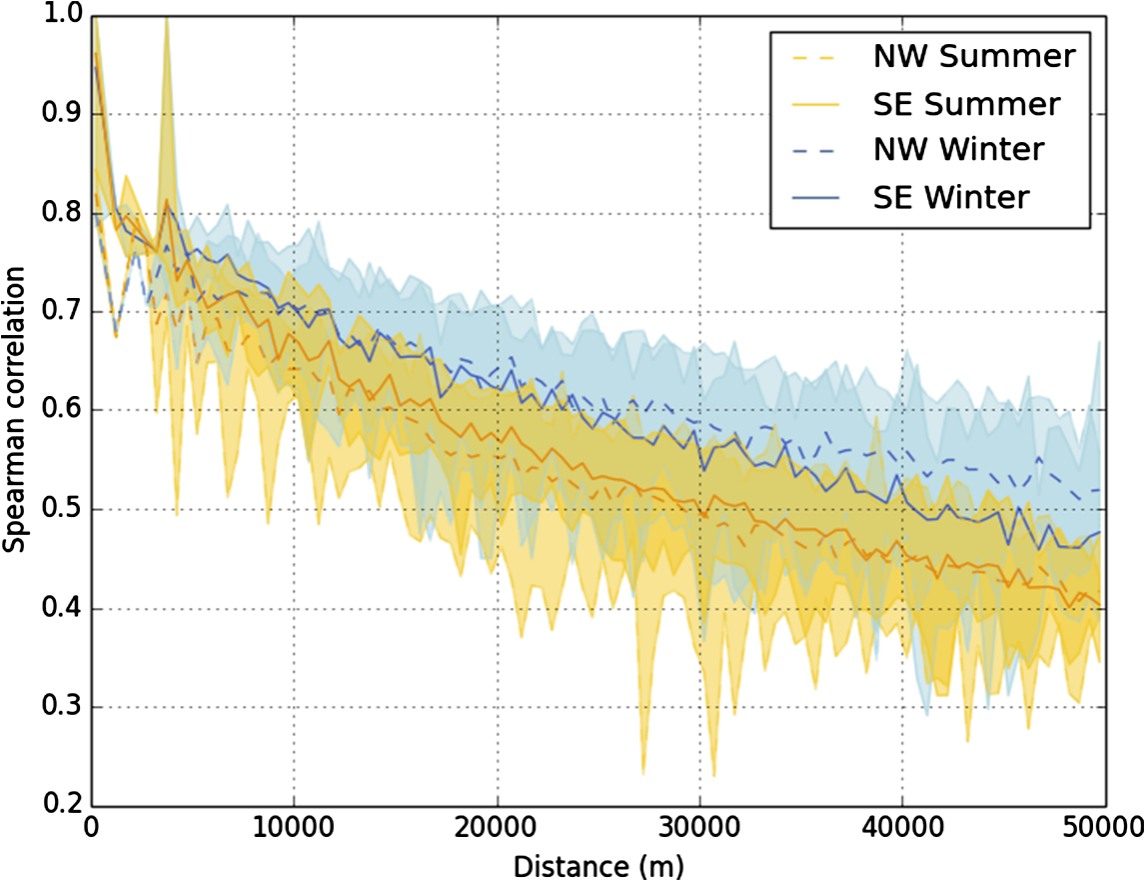
Winter (DJF) and summer (JJA) Spearman correlation (r_s_) of hourly time series with distance. Correlations were calculated separately using an arbitrary NW–SE regional division. Lines show average r_s_, range show maximum and minimum correlation.

There is no standard approach for the QC of sub‐daily rain gauge data. A suite of procedures are therefore used here (summarized in Table 2 and outlined below), primarily focussing on constructing a high‐quality dataset for the assessment and application of extreme sub‐daily rainfall but going beyond the FEH approach (Faulkner, 1999) of focussing on AM, expanding the QC process so that other potential artefacts in the data may be identified. These procedures are single rain gauge tests where each gauge is considered independently. Multiple rain gauge tests, which compare gauges with neighbours, offer further advantages but are challenging for the assessment of sub‐daily extremes. A comparison of the winter (DJF) and summer (JJA) correlations between 100 randomly sampled gauges (after single rain gauge QC tests were applied) indicates a more rapid decrease with distance in summer, at a distance of <10 km, compared with winter (Figure 3), reflecting the more localized nature of rainfall in JJA (Hand *et al.*, 2004). This presents difficulties in using neighbouring gauges as checks in summer, particularly for the QC of extreme values. The use of such approaches is therefore the subject of ongoing work, and so, these are not employed here.

**Table 2:** Summary of main QC procedures applied to all data and described in the main text.

Acronym	Description
Rainfall amounts	
QC1*	Hours UK 1 h record exceeded by ≥20%
QC2	Hours where 80% of 1 h record exceeded in April–October period
QC3*	Days UK 24 h record exceeded by ≥20%
QC4*	Hours where suspect ‘large’ daily accumulations at 0900
QC5*	Hours where suspect consecutive daily accumulations at 0900 or 1200 (at least 3 consecutive days or 2 days where totals > trace amounts of 0.2 mm)
QC6	Hours where suspect monthly accumulations
QC7*	Hours with consecutive ‘large’ values
QC8*	Hours identified as frequent tipping [applied only to EA TBR data using the Upton and Rahimi (2003) algorithm]
Dry periods	
QC9*	Suspect ‘terminal’ dry spells at start/end of gauge record ≥1 month duration
QC10	Dry periods ≥1 month duration
QC11*	Dry periods ≥45 days duration

The acronyms are used in the text and in Figure S2. Those marked by an asterisk denote those that are used to automatically signify suspect data; those without an asterisk were only considered suspect if multiple tests produced a flag or in the presence of additional supporting evidence in the metadata.

#### 2.3.1 Identification of TBR high frequency tipping

One specific problem noted with EA TBRs is that of high‐frequency tipping, which leads to the recording of spurious high rainfall intensities. Such problems may arise due to a mechanical malfunction, or where gauges are unheated, such rapid tipping may be associated with thawing snow (Upton and Rahimi, 2003). This may result in large hourly accumulations that are readily identifiable (Faulkner, 1999). However, for relatively short‐lived periods of high‐frequency tipping, an alternative detection method is required. Here, the method described by Upton and Rahimi (2003) was used. Their method is based on the assumption that rainfall rates change slowly, and so, the evaluation of the statistic *λ_k_* is given by:

(1) λk=|lnτkτk−1|

where *τ_k_* is the inter‐tip time. They identify λ*_k_* > 5 as a threshold for the rejection of a tip. Sequences of at least 10 high‐frequency tips (τ*_k_* < 5 s; Upton and Rahimi (2003)) following the exceedance of this threshold were therefore identified and treated as suspect (QC8, Table 2) unless they had been subject to verification against a check gauge by the EA and marked as good.

#### 2.3.2 Identification of erroneous and accumulated totals

After the TBR data had been accumulated to hourly totals, the combined dataset from all three sources was subjected to a further sequence of independent, single rain gauge checks. Firstly, potentially erroneous hourly values were identified using a number of procedures and examined. These may arise as simple recording errors or may be values derived from accumulations over longer time periods. It is important to note that some of these procedures should not be considered independently but be used to provide accumulated evidence for potentially suspect hourly totals. We therefore sub‐divide the tests into two categories:

1. Values automatically treated as suspect
  - A small number of negative values mainly occurring as a consequence of erroneous coding of missing data were removed and replaced with correct missing data markers.
  - The record UK 1 h rainfall total is reported as 92 mm (see http://www.metoffice.gov.uk/public/weather/climate‐extremes/#?tab =climate Extremes for a list of UK climatological extremes). However, this does not necessarily mean that larger totals identified in this dataset are erroneous as it comprises a larger network of gauges and therefore may capture additional, localized intense events. All hourly totals exceeding this amount by 20% or more were identified and treated immediately as suspect (QC1). In this way, obvious recording errors, such as incorrect coding of missing data, recording malfunctions, persistent high‐frequency tipping and obvious accumulations over longer periods, could be quickly highlighted and denoted as suspect. The same process was applied to daily (24 h) totals using the corresponding record of 279 mm, which was compared with accumulated hourly rainfall over the period 0900–0900 (QC3). Hourly/daily totals exceeding these records by less than 20% were further assessed by checking against the raw TBR data and metadata (where available) and along with all other data by using the additional checks described here and in list 2).
  - Daily (24 h) accumulated values were noted to be most likely to occur at 0900 and 1200 and were identified using two procedures. Firstly, hourly totals at 0900, which exceed two times the mean daily rainfall intensity for that month, *and* follow 23 h without any recorded rainfall were treated as suspect (QC4) along with the preceding dry period. Secondly, as daily accumulations were noted to occur in continuous sequences, consecutive daily rainfall occurrence at 0900 and 1200 (at least 3 days with no threshold applied or 2 days where both exceed trace rainfall measurements) *and* following 23 h without recorded rainfall were also treated in the same way (QC5).
  - Duplicate rainfall totals in consecutive hours (exceeding two times the mean daily rainfall intensity for that month) were recorded as suspect (QC7).

Values are only treated as suspect if additional evidence is available either from raw TBR data and metadata or flags from multiple QC tests:
  - As the most intense 1 h rainfall was subsequently noted to generally occur in summer and early autumn, values in the period October to April exceeding 80% of the record 1 h total were flagged (QC2).
  - Potential monthly accumulated values were identified as months with only one hourly value in excess of two times the mean daily rainfall intensity for that month (QC6).
  - Specific examination was also undertaken of intense events that occurred frequently at specific dates [e.g. first/last day of the month, Boxing Day (26 December)].

In the absence of multiple sources of evidence, hourly totals identified by only one test in list 2 were retained to avoid legitimate values being discarded. Manual inspection of TBR data and metadata is a time‐intensive process and so was limited here to the final gauge selection described below based on data length and completeness. This included additional strategies of *ad hoc* comparison with nearby gauges and documentary sources of information such as the Royal Meteorological Society's *Weather Log* and the event chronology provided in Eden (2008). The relative frequencies of potential QC issues for each data source is summarized in Figure S2, which, as expected, demonstrates that the extent of flagged suspect data reflects the rigour of previous QC (few erroneous/suspect data are identified in the MIDAS rain gauges) and that each data source is affected by different issues. For example, a substantial number of daily accumulated values were identified in the SEPA gauges. The effect of these additional QC procedures on indicators of the intensity and frequency of 1 h extremes used in the subsequent climatological analysis [median AM (*R*_med_) and Extreme Rainfall Alerts (ERA) events respectively as described below] is provided in Figures S3 and S4. This demonstrates that whilst these statistics are only affected for a minority of rain gauges (up to ∼20% of JJA *R*_med_ values are revised following the additional QC), the measures taken are essential to avoid erroneous analysis of some features of local and regional climatological and hydrological extremes.

A further important point to note is that the QC of observed data should be considered a multi‐facetted process. Ideally, the above checks should be combined with local/regional knowledge of climatological processes and basic climatological data analysis. For example, the subsequent assessment of extremes detailed in this paper identified a significant seasonality in hourly extremes in the UK linked with rainfall generating processes that could subsequently be used to identify and investigate totals that were not consistent with this knowledge, for example, intense events occurring in winter. Such process‐based knowledge may therefore contribute to QC procedures.

#### 2.3.3 Identification of erroneous dry spells/gauge non‐operation

Although this research is focused on assessing the sub‐daily extreme rainfall climatology of the UK, it was also necessary to undertake some assessment of dry spells in the rain gauges as not all gauge malfunctions are readily identifiable in the TBR data, which may lead to the erroneous recording of zero values and indicate that a gauge is suitable for analysis over a given period when in fact this is not the case. The identification of the most egregious errors in terms of dry spells in the record is important as failure to do so may lead to seasonal and annual statistics being derived from unrepresentative, small samples. Unfeasibly long dry spells (e.g. > 6 months) were identified for some rain gauges and could be readily treated as missing. In some instances, significant dry sequences occurred at the start of the record, suggesting that the gauge start date had been incorrectly recorded and populated by zero rainfall values. In other cases, such spells were noted at the end of the gauge record, which suggested that it had ceased to operate some time prior to the termination of the record. Such ‘terminal’ dry spells were treated as missing data (QC9). All other instances where no rain was recorded (‘non‐terminal’) for periods of greater than 1 month were flagged for further investigation (QC10), with an additional flag to identify the most extreme spells of longer than 45 days (QC11; see Figure S2), alongside the percentage correct statistics described below.

### 2.4 Assessment of consistency with a quality‐controlled daily precipitation dataset

**Figure 4:**
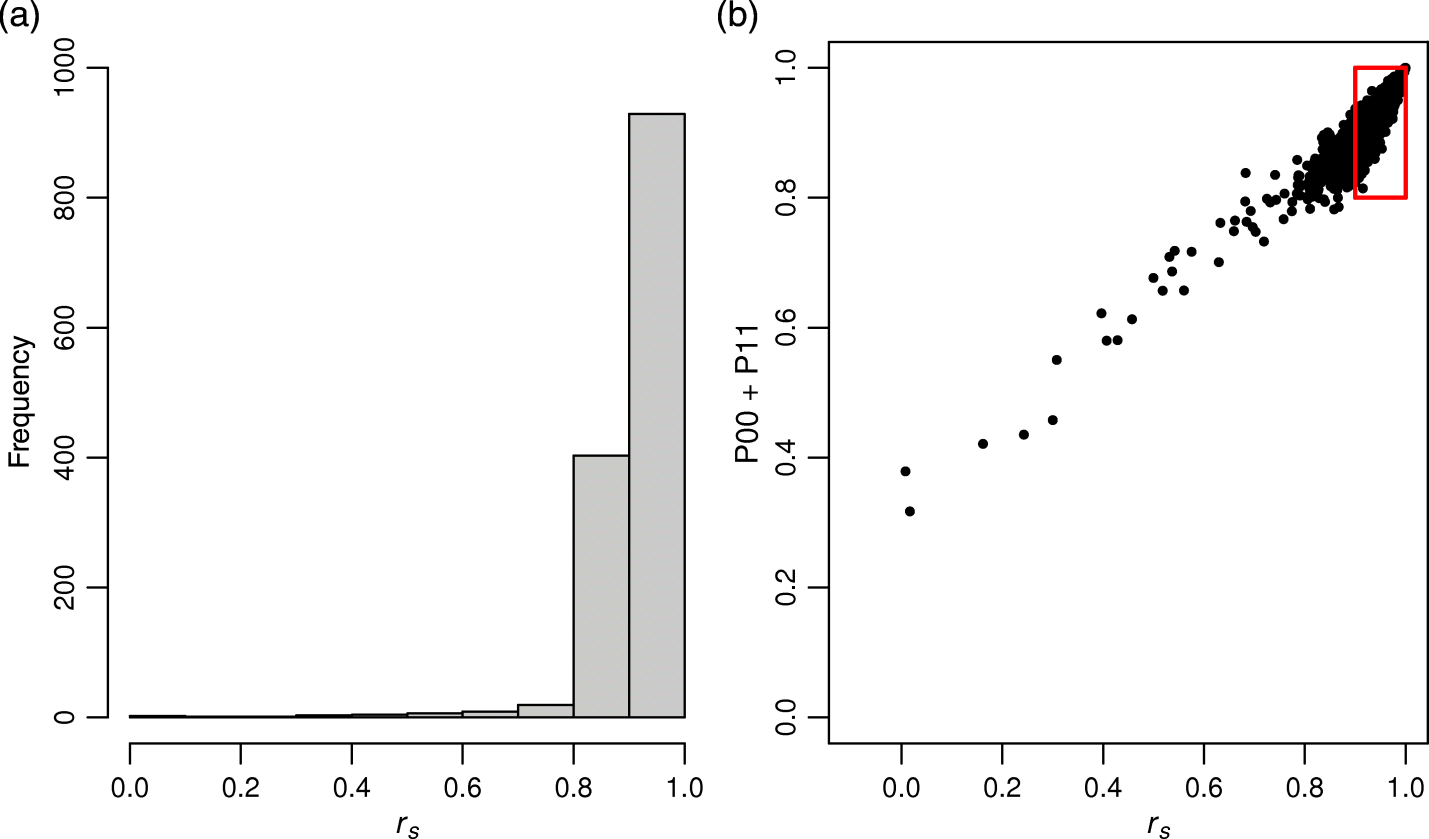
(a) Frequency distribution of r_s_ for each gauge and corresponding 5 km grid square and (b) comparison of r_s_ and percentage correct statistics for each gauge before gauge exclusion as detailed in the text. The box denotes the area within which gauges were considered acceptable for inclusion.

As a check on the overall quality of the data, the hourly records for each gauge were accumulated over the period 0900 on the day recorded to 0900 the next day and compared with the UKCP09 5 km gridded daily rainfall dataset (Perry *et al.*, 2009). This dataset was created from the Met Office archive of daily observations and covers the UK over the period 1958–2006. These gauge data have already been quality‐controlled and have additionally been inspected for obvious errors. For each hourly rain gauge, the accumulated daily total was compared with the corresponding grid square in the UKCP09 dataset for the period, for which they are coterminous. Spearman's rank correlation coefficient (*r_s_*) was used to highlight errors in the hourly dataset by comparing each rain gauge and its corresponding 5 km grid cell. Figure 4(a) shows the distribution of correlation values for each gauge‐grid cell pair, demonstrating a high correlation for most gauges. Gauges with an *r_s_* of less than 0.9 were investigated, often revealing a very obvious error that could provide further evidence for the single gauge QC checks.

‘Percentage correct’ statistics, often used by the forecasting community, were also used here to assess the reliability of rainfall occurrence in the rain gauges. These count the proportion of days on which it rains in both records (P11), the proportion of days in which it rains in the daily record but not the aggregated hourly record (P10), the proportion of days that it rains in the hourly record and not the daily record (P01) and the proportion of days where it does not rain in either record (P00). P00 + P11 therefore shows how concordant the two records are. Wilks (2006) indicates that this test may not be appropriate when event occurrence is rare as event non‐occurrence is then easy to predict (correctly match). This limitation does not constrain its use in the context of UK rainfall occurrence, but we note that this statistic may not be readily transferable to some climate regimes, and other methods reviewed by Wilks (2006) may be more appropriate. Figure 4(b) shows a comparison of the matching statistics with *r_s_* values for each rain gauge. Gauges with high P00 + P11 but low *r_s_* tended to be those with spurious large values, whilst those with low P00 + P11 and low *r_s_* were those with extended dry sequences. After the replacement of all suspect data identified from this and the other additional QC tests with missing values, rain gauges whose *r_s_* was less than 0.9 or with P00 + P11 < 0.8 were automatically excluded.

### 2.5 Summary of available data

**Figure 5:**
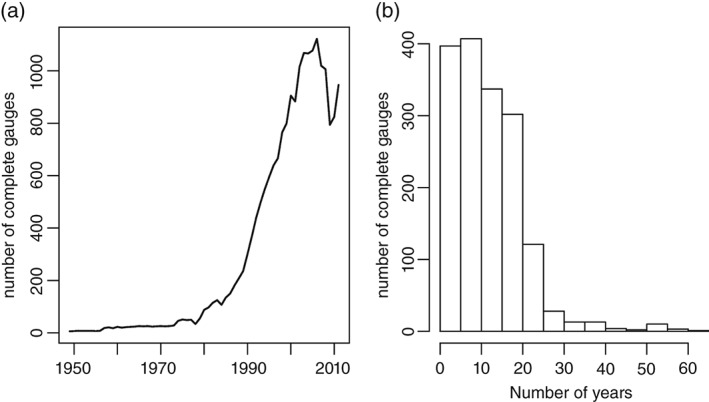
(a) Number of rain gauges providing ‘complete’ years of data and (b) the frequency of rain gauges contributing different numbers of ‘complete’ years.

The total dataset comprises 1638 gauges, and as shown in Figure 5(a), the gauge density only increases substantially from the mid‐1980s, with most gauges (particularly the EA TBRs) commencing in the early‐ to mid‐1990s. A ‘complete’ gauge is defined here as one with no more than 15% of hourly data missing in a given year and is therefore considered available for climatological analysis. The maximum number of ‘complete’ gauges is 1122 in 2006, but this decreases thereafter to 794 and 824 in 2009 and 2010, respectively. This decrease is largely due to a greater amount of missing data across the gauge network in winter 2008/2009, 2009/2010 and December 2010. Figure 5(b) shows that there are relatively few contiguous gauges in the network, with ∼750 gauges (mainly EA TBRs) contributing less than 10 ‘complete’ years of data, whilst 567 (259) gauges contribute at least 15 (20) ‘complete’ years. The longest records are from the MIDAS dataset (earliest record 1949), whilst those from SEPA and the EA start later (earliest records 1981 and 1962 respectively, although the first ‘complete’ year of data is 1982 and 1965 respectively).

### 2.6 Analysis of hourly extremes

A brief analysis of the climatology of hourly extremes is made to provide basic information on the characteristics of UK intense rainfall, principally the magnitude and timing of events and their spatial variability. The aim of this analysis is firstly to provide a descriptive climatology of extremes and to also provide an additional ‘sanity test’ of the new dataset, which links the observed climatology to known processes. As this is an initial application of the new dataset, three relatively simple methods are used to provide this climatology.

- For each gauge, seasonal AM were calculated from which the R_med_ statistic (median AM) was determined. R_med_ corresponds to an RP event of 2 years (on the AM scale) and is robust to outliers in the AM time series. It has been used to assess changes in UK daily rainfall extremes (Fowler and Kilsby, 2003a, Jones et al., 2013) and is also used as an index variable in the FEH (Faulkner, 1999) as it can be reliably estimated from the record lengths typically available for UK sub‐daily rainfall (Stewart et al., 1999). It is used in the FORGEX (Focused Rainfall Growth Extension) method, which estimates rainfall totals with long RPs (Reed et al., 1999) and which, together with the FEH, provides a standard methodology for rainfall frequency estimation for the hydrological community in the UK. Events with a longer RP are required in the design of hydrological infrastructure, for example, urban drainage systems (e.g. WRc, 2006; Dale et al., 2015) and extreme value statistics may be used to assess the climatology of such events. This has been applied to daily rainfall for the UK (e.g. Rust et al., 2009) and daily and multi‐day rainfall as part of regional frequency analysis (e.g. Fowler and Kilsby, 2003a; Jones et al., 2013). The use of such methods to identify long RP events goes beyond the scope and capacity of this paper but forms part of the ongoing analysis of this new dataset.
- Extremes defined using an n‐largest approach where the largest n.m events are selected, where m is the length of record in years. In contrast with AM, more than one event may be sampled from a given year and none sampled from another. For example, for n = 1, a complete 20‐year record would identify the 20 largest hourly totals across the whole record (hereafter referred to as n1 events); for n = 3, the largest 60 events would be considered (hereafter n3) etc. It thus provides more comprehensive information than the AM series as all extreme events may be represented. The declustering algorithm described by Ferro and Segers (2003) was used to identify independent rainfall events. This is a statistical approach that estimates an extremal index based on the event inter‐exceedence times to represent the proportion of these times that may be regarded as being between independent clusters. The declustering algorithm is applied here with a threshold of the 95th percentile wet hour amount for each rain gauge. This ensures that events are excluded if they are not ‘extreme’, although this constraint only affects a small number of rain gauges for n5 events, that is, the declustering process generally provides at least 100 events for the analysis of n5 events. The method was implemented using the R package POT (Ribatet, 2012) to provide a means for the objective determination of independent events, with the highest hourly value within a cluster used for each event.
- A practical indicator that relates extreme rainfall climatology to flood risk is the series of thresholds prescribed by the operational ERAs, which provide warnings of extreme rainfall based on intensities that are likely to cause severe surface water flooding in urban areas (Hurford et al., 2012a). These were defined by Halcrow (2008) to approximate the 1‐in‐30 year RP FEH rainfall intensities (Faulkner, 1999) for eight UK cities, and the 30‐year RP is used as the design standard for most urban drainage systems in the UK (WRc, 2006).

To assess the spatial characteristics of the UK extreme hourly rainfall climatology, we also use the regional classification developed by Jones et al. (2014). Although this was derived based on the characteristics of daily rainfall extremes, such as magnitude, timing and variance, we consider this a reasonable classification to initially explore the spatial pattern of hourly extremes.

## 3 Results

### 3.1 A climatology of UK hourly extremes

To ensure that the dataset describes the climatology of extremes well, gauges with a sufficient record length are required. However, imposing a requirement for too long a record limits the available data and the capacity for meaningful analysis. Given the low number of gauges with a span exceeding 20 years (see Figure 5(b)), only those covering the period 1992–2011 were used (maximizing the number of available gauges of this duration). This is sufficient to provide a robust climatology of the measures of extremes described above. Gauges were excluded from the analysis if more than 15% of all years/seasons over this period were incomplete as defined earlier. As a result, 376 gauges were available for analysis for this period (Figure 1(b)), although due to variations in the amount of missing data, the number of usable gauges available for seasonal analysis varies: annual (ANN) – 192; winter (DJF) – 215; spring (MAM) – 255; summer (JJA) – 212; autumn (SON) – 268. Figure 1(b) indicates that the MIDAS gauges are relatively evenly distributed across the UK, but the EA TBRs are relatively sparse in parts of Wales, eastern England and, particularly, SW England, largely as a consequence of the varying times at which different administrative regions were instrumented. The earliest records in the EA's Southwest region begin in 2000, compared with 56% (28%) beginning in 2000 or later in their Anglian (Southeast) region.

**Figure 6:**
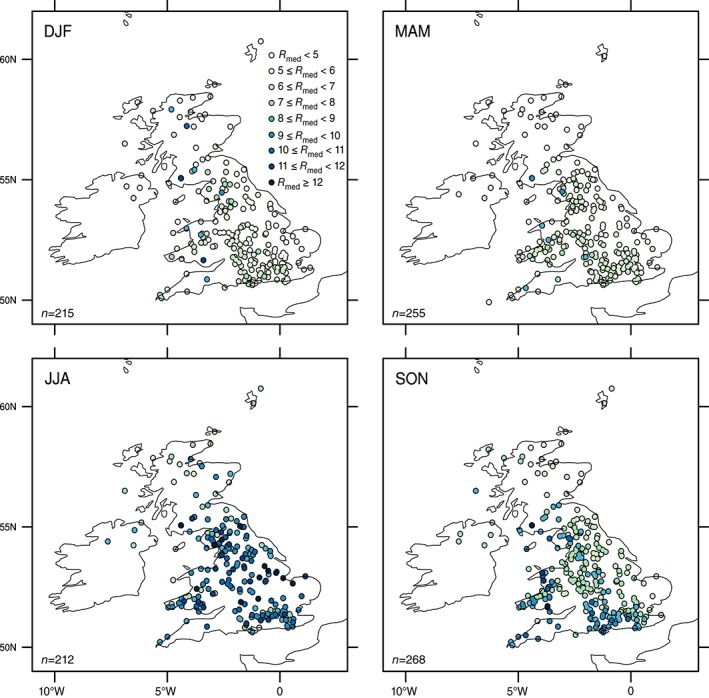
Seasonal 1 h R_med_ (in mm) for the period 1992–2011, n denotes the number of gauges for each season.

Analysis of the seasonal 1 h R_med_ is provided in Figure 6, indicating that in winter there is an east–west, orographically influenced pattern of rainfall, a pattern that is also apparent in autumn, although with a reduced gradient. In spring, the pattern is more northeast–southwest, with higher intensities extending across central and southern England. This is consistent with that identified for 1 d extremes by Maraun et al. (2009), although their study determined that the heaviest precipitation typically occurs during late autumn and winter along the west coast, whilst along the east coast and the Midlands, late summer is generally associated with extremes. Here, the highest hourly R_med_ intensities occur in summer when the spatial pattern is much less well pronounced, although higher values are observed in England and Wales and the lowest in northwest Scotland.

These seasonal patterns are likely to be a consequence of the rainfall generating‐mechanisms operating at different temporal scales at different times of the year. For longer accumulation periods, synoptic scale systems are responsible for extreme events, but at 1 h durations, local‐scale convective events are more important in summer (Hand *et al.*, 2004), particularly in the south and east. This is consistent with the high convective available potential energy (CAPE) in this region (Holley *et al.*, 2014) and with thunderstorm climatologies for the UK, which have indicated greater storm incidence in the southeast part of the UK (Holt *et al.*, 2001; van Delden, 2001). Some of the highest intensities are also observed over the uplands of northwest England and Wales (Figure 6), suggesting that in some regions, there may be multiple influences. Examples provided in the supporting information show consistent spatial patterns for mean wet hour intensities (MWHI, mean of all wet hours – rainfall ≥0.2 mm; Figure S5) and 99th and 99.9th quantiles (Figures S6 and S7).

Events defined by the operational ERAs were also examined, using the 30 mm h^−1^ threshold used for 1 h totals. Fewer than 60 such events (>50% occur in JJA) are recorded in this dataset (Figure S8), which, coupled with their highly localized nature, makes it difficult to analyse their spatial occurrence based on measurements at discrete points afforded by a rain gauge network. However, although Hurford *et al.* (2012b) note that surface water flooding is associated with rainfall at lower intensities and with shorter RPs, they do provide a simple link between rainfall and flash flooding that may be used to understand important atmospheric drivers of such events and provide information on when risk is greatest.

**Figure 7:**
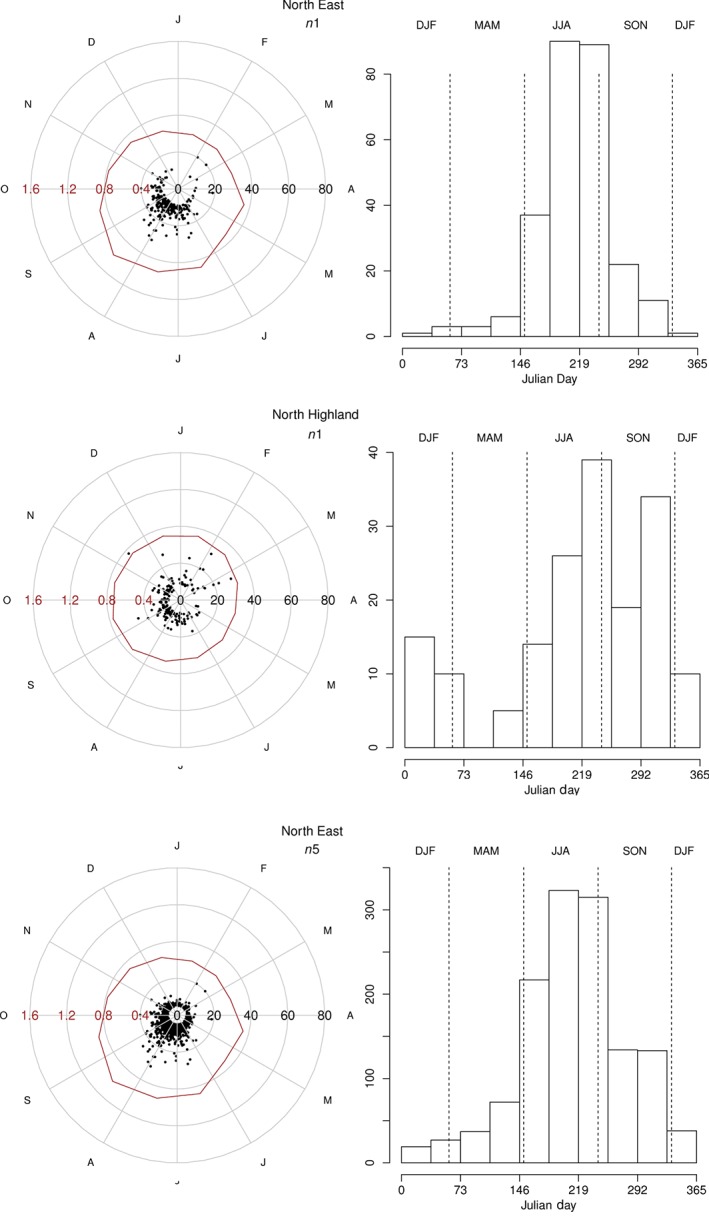
Radial plots (left column) for North East n1, North Highland n1 and North East n5 (where n1 and n5 represent the 20‐, and 100‐largest events, respectively, in a complete 20‐year record). Black dots identify events with magnitude denoted by the right axis (in mm). Monthly MWHI is shown by the solid line denoted by the lef axis (in mm). Histograms (right column) show frequency of daily occurrences of n‐largest events by Julian Day, with seasonal divisions shown by the vertical lines.

The seasonality of intense rainfall events is assessed in greater detail using the circular statistics employed by Robson and Reed (1999) and applied here to events determined using the *n*‐largest approach. This method (see Appendix S1 for complete details) yields a mean day of year for events, $\bar{\theta }$, and the concentration of the seasonal distribution, $\bar{r}$. A value of the latter close to 1 indicates that events usually occur at the same time of the year, whereas a lower value indicates that seasonality is weaker, and consequently, the value of $\bar{\theta }$ is less representative of the distribution. Values of $\bar{\theta }$ and $\bar{r}$ for each of the extreme rainfall regions are shown in Table 3. The occurrence of *n*1 events has the strongest seasonality in eastern regions – North East, East Anglia, Humber, Forth and South East – $\bar{r}$ ≥ 0.65. For these regions, the peak time of year is also associated with the highest magnitude events as shown in Figure 7 for *n*1 events for the North East region (14 gauges). In contrast, the weakest seasonality occurs in western regions and northern Scotland as illustrated by the North Highland region (nine gauges, $\bar{r}=0.41$). The associated histogram clearly demonstrates the higher relative frequency of events in autumn and winter in this region. The annual distribution of *n*1 events for all regions is shown in Figure S9. The mean time of occurrence, $\bar{\theta }$, of *n*1 events is in summer for all regions except North Highland, for which mean occurrence is in mid‐autumn, ∼50 days later than the earliest regional mean occurrence. These characteristics broadly reflect the monthly MWHI, also shown in Figure S5, which in the southern and eastern regions is at a maximum in late summer and is lowest during winter and early spring. The evidence presented by Rust *et al.* (2009) suggests that this seasonality is also preserved in southeast England daily rainfall totals to some extent. As additional events (of lower intensity and typically non‐convective in origin) are incorporated by examining *n*3 and *n*5, the mean day of occurrence becomes progressively later in all regions (to autumn for a number of northern and western regions) as these are more likely to occur in autumn (see also Figure 7 for North East).

**Table 3:** Circular statistics for rain gauges in each rainfall region for n1, n3 and n5 events (the 20‐, 60‐ and 100‐largest events, respectively, in a complete 20‐year record).

	$\bar{\theta }$			$\bar{r}$		
	*n*1	*n*3	*n*5	*n1*	*n*3	*n*5
North East	*217*	*218*	*218*	0.77	0.63	0.56
East Anglia	*215*	*219*	*222*	0.72	0.61	0.55
Humber	*209*	*215*	*216*	0.71	0.60	0.53
Forth	*228*	*236*	*235*	0.67	0.53	0.48
South East	*221*	*229*	236	0.65	0.53	0.45
North West England	*216*	*228*	*235*	0.61	0.45	0.38
Mid North Ireland	*227*	*236*	*242*	0.60	0.50	0.41
West Country	*222*	*233*	*241*	0.60	0.50	0.41
South West	*235*	250	260	0.55	0.46	0.40
Solway	*238*	244	252	0.52	0.47	0.41
East Scotland	*222*	*230*	*236*	0.52	0.43	0.38
South Scotland	*240*	252	255	0.51	0.46	0.42
Mid and North Wales	*220*	*233*	245	0.51	0.39	0.31
North Highland	260	269	278	0.41	0.37	0.33

Statistic $\bar{\theta }$ denotes the mean event day of the year (Julian day); $\bar{r}$ indicates the degree to which events are seasonally concentrated. Italicized values of $\bar{\theta }$ indicate a mean occurrence in summer; all other values are in autumn (in a non‐leap year, the climatological summer is between days 152 and 243). Regions are ranked in descending order of *n*1 $\bar{r}$.

**Figure 8:**
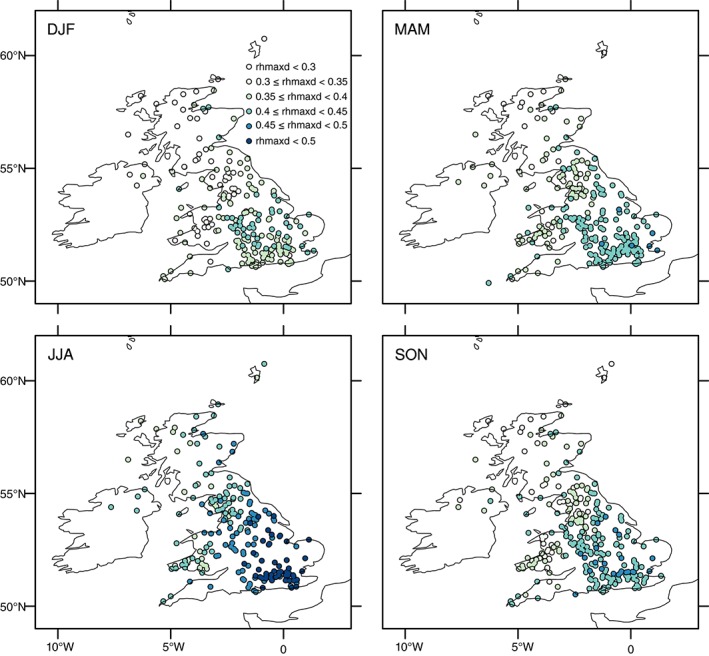
Mean seasonal values of the ratio between 1 h maximum rainfall and 1 d rainfall (rhmaxd) for the period 1992–2011.

**Figure 9:**
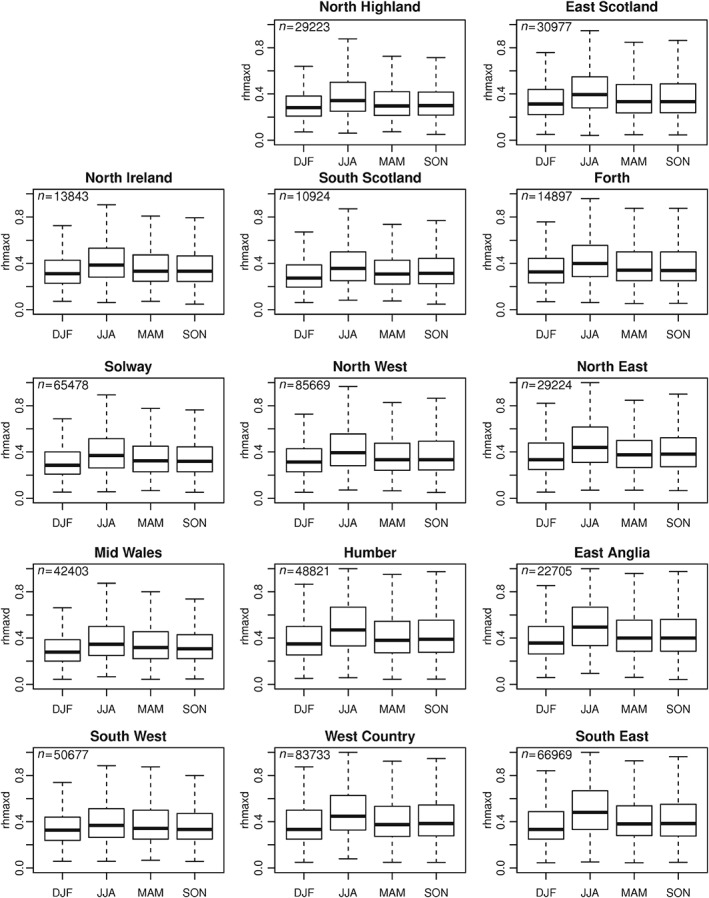
Seasonal distribution of the ratio between 1 h maximum rainfall and 1 d rainfall (rhmaxd) for the period 1992–2011 for each of the extreme rainfall regions. The value n denotes the number of gauge wet days per region.

A useful index for assessing the daily distribution of rainfall is the ratio between the maximum 1 h and total 1 d accumulation (e.g. Westra *et al.*, 2013). Here, we examine the ratio (hereafter referred to as rhmaxd), for each wet day (defined as each day where rainfall is at least 1 mm to eliminate the effect of small rainfall amounts). Values approaching 1 indicate rainfall occurring over only a small portion of the day, whilst those approaching 0 represent constant rainfall occurring over the 24‐h period. Here, for each gauge the ratio is calculated as the mean from all wet days and is presented seasonally in Figure 8. As expected, summer has the highest ratio of maximum 1 h bursts, with peak values >0.5 in the south and east, decreasing to <0.4 in northwest Scotland. These higher values reflect the more frequent occurrence of intense, short duration, convection‐driven storms. Similar spatial patterns are present in other seasons but values are lowest in winter, from <0.3 in northwest Scotland and parts of Wales up to ∼0.45 in central and eastern England. Similar patterns are also observed when the analysis is repeated to examine only days exceeding the seasonal 95th percentile daily rainfall total (not shown). The spread of values of rhmaxd is shown in the box plots for each of the rainfall regions in Figure 9. This suggests that the increase in the summer index is greatest in the south and east – shown by the median and also the extremes of the distribution but also highlights the large variability in this ratio, which reflects the different types of rainfall events across the UK.

**Figure 10:**
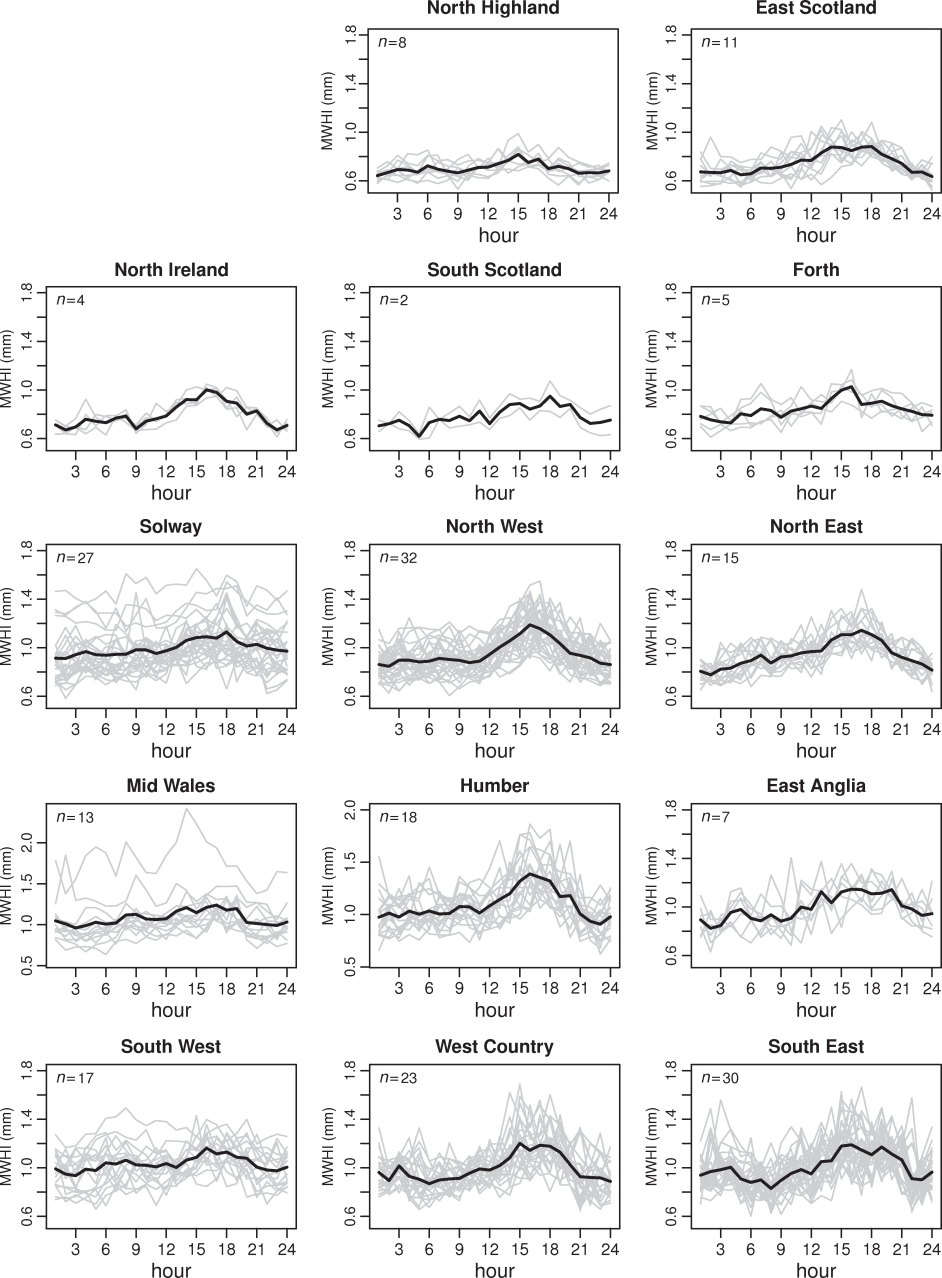
Mean summer (JJA) wet hour intensity (MWHI) for each extreme rainfall region (bold lines). Grey lines show mean intensities for individual gauges. Note altered vertical scale for Mid Wales and Humber. The value n denotes the number of rain gauges per region.

The diurnal cycle of rainfall was initially examined for each gauge using the MWHI for each hour from which hourly means for each extreme rainfall region were calculated. For summer, over half of the regions possess a clear diurnal cycle (Figure 10), with peak MWHI in the mid‐ to late afternoon. Regions without a diurnal cycle in MWHI tend to be in the west, although the spatial pattern is not clearly defined, with a strong cycle in North West England. There is also clear variability within some regions as a consequence of local‐scale climatic influences. This diurnal cycle is also apparent in a small number of regions in autumn, although of a much smaller amplitude and is absent in spring and winter (not shown).

**Figure 11:**
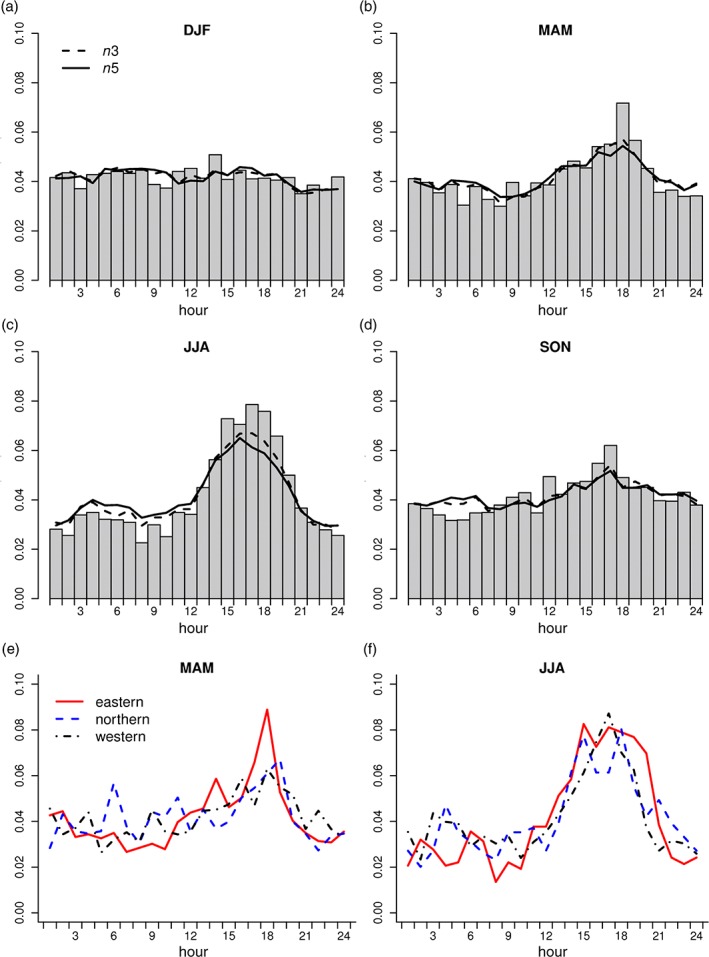
Frequency distribution of seasonal n1 events by hour for (a) winter, (b) spring, (c) summer and (d) autumn. Data are pooled for the whole dataset, and n1 frequencies are indicated by bars; n3 and n5 events by lines (the 20‐, 60‐ and 100‐largest events, respectively, in a complete 20‐year record). Regional frequencies are presented for (e) spring and (f) summer for three amalgamated regions as defined in the text.

The diurnal cycle for extreme hourly rainfall was then examined for *n*1 events (Figure 11(a)–(d)), this time using the *n*‐largest approach separately for each season. Individual extreme regions were not used in this instance as some contain relatively few gauges and so do not provide sufficient data to sample across the 24‐hourly bins. These events were therefore initially examined by pooling all rain gauges, again showing a clear seasonal dependency. Hourly extreme events are evenly distributed throughout the day in winter, reflecting the large‐scale systems that produce such rainfall, which are not time dependent. In contrast, summer events show a clear peak between 1400 and 1900, suggesting a dependency on convection associated with higher temperatures, whilst there is a much lower amplitude in the diurnal cycle in the transitional seasons. The same features are also apparent for *n*3 and *n*5, although the amplitude of the summer diurnal cycle is slightly lower when additional, less intense totals are included.

A brief assessment of the spatial consistency of the diurnal cycle of hourly extreme rainfall across the UK was obtained by examining the same data for three larger regions derived from the amalgamation of the extreme rainfall regions. An eastern region reflecting the area identified previously as having the strongest seasonality for *n*‐largest events, was constructed from regions 3, 4, 6, 8 and 10 (comprising 75 gauges in JJA) in Figure 1. A northerly region reflecting an area with lower magnitude of summer extremes was also constructed (regions 9, 11–14; 52 gauges) along with a western region (regions 1, 2, 5 and 7; 85 gauges). Figure 11(e) suggests that the diurnal cycle of hourly rainfall extremes is strongest in the eastern region in spring and somewhat less pronounced elsewhere, particularly in the northern region, the same being true for autumn (not shown). In summer (Figure 11(f)), there is less regional variation with the peak frequency of *n*1 in the eastern region possibly extending later into the day. For *n*5, there are a sufficiently large number of events to examine the diurnal cycle of hourly rainfall extremes for individual extreme rainfall regions. This is not shown for conciseness, but for some western and northern regions (South West, Solway and North Highland), the summer diurnal cycle is not present, most likely as a consequence of the mixed origin of events with the inclusion of low intensity, non‐convective rainfall.

## 4 Discussion and conclusions

A new dataset of hourly rainfall for the UK has been produced, combining three different sources of rain gauge data. As with all observational datasets, QC is an essential process before data analysis and wider application. This paper has demonstrated that there may be significant problems associated with raw TBR data, which in the UK, have generally been collected for operational functions rather than for long‐term climatological analyses. We therefore describe a series of QC procedures that may be applied to both TBR data and data accumulated to hourly totals. The quality of the data is checked by accumulating the hourly values to a daily total for comparison with a quality‐controlled gridded daily rainfall product, and only those showing good agreement are retained. These QC procedures focus on the identification of spurious extreme values and may form the basis of a set of standard checks for sub‐daily data. They are not expected to produce a ‘perfect’ dataset, but we consider that they provide a reliable one for the climatological analyses of extreme hourly rainfall. The methodology is best equipped to identify spurious extreme rainfall amounts, but additional procedures may be required for an assessment of moderate rainfall amounts and dry spells. Such additional work may be required before the data is suitable to drive hydrological models for some types of analyses. We consider the next steps therefore to be:

1. The development of methods for the use of neighbouring gauges as an additional check of hourly totals and dry spells. This is however a non‐trivial issue, particularly considering the localized nature of summer storms.
2. The development of a ‘rule base’ that may be used to combine the procedures described in this paper with (1) above to provide an automated QC process requiring minimal manual data checking. This would enable the efficient assessment of the considerable amount of data before 1992 in this dataset.
3. The application of additional assessments of long‐term homogeneity through the use of appropriate statistical tests for break points are required before the analysis of longer‐term trends and variability in sub‐daily rainfall. As Figure 5 shows, there are ∼40 gauges extending longer than 30 years that could be used for such analysis.
4. The determination of appropriate statistically defined thresholds that may be used to identify the most egregious errors and accumulations in different climatic regimes globally, particularly where national records are not available or for countries with large geographical climatic variability where a single record total may be less appropriate.

A subset of this data was applied to assess the climatology of hourly extremes for the UK, comprising 376 gauges that were near‐complete for the period 1992–2011. AM and percentile approaches were used seasonally to identify a west–east pattern of decreasing extremes from autumn through to spring. This is similar to that observed for daily rainfall accumulations, which are influenced by the prevailing direction of cyclonic weather systems and modified by orography, western and northern areas being associated with orographically enhanced rainfall. In summer this pattern of extremes is replaced by a less well‐defined north–south pattern, when extreme hourly rainfall is less likely to be associated with such mesoscale weather systems and more probably a consequence of local‐scale convection (or convection embedded within such systems).

The climatology also indicates that, as well as the highest intensities, summer is associated with the most frequent occurrence of hourly extremes across the UK. This seasonality, examined primarily using the *n*‐largest events, is most pronounced in eastern coastal regions of England and Scotland and suggests that the risk of flash flooding in urban areas and rapid response river catchments may be greatest in summer in most parts of the UK. However, the spatial distribution of ERA events (using the 1 h threshold as shown in Figure S8), which also predominantly occur in summer, albeit representing relatively rare events, indicates fewer of the most intense totals in eastern coastal regions. This seasonality has some similarities to the analysis of daily rainfall extremes presented by Rodda *et al.* (2009) using data from the observers network published in *British Rainfall,* which shows most events occurring from July through to December, although the most extreme events (>150 mm per day) are noted to be bimodal, with peaks in summer and winter (this may have been a consequence of failing to distinguish between different precipitation regimes across the UK). However, the seasonality identified here has a limited similarity to that noted for daily rainfall by Rust *et al.* (2009), although direct comparison is difficult as theirs was based on the magnitude of 10‐ and 100‐year daily rainfall events. They showed that for daily events, the autumn and winter dominance of orographically enhanced frontal rainfall is more spatially extensive along the west coast of the UK than is indicated by the hourly analysis presented here. Furthermore, the pattern of higher magnitude summer extremes is also less spatially extensive at a daily timescale, with high summer and lower winter return levels noted only in the southeast UK. As noted earlier, the application of extreme value analysis to this dataset would prove extremely useful, including comparative analyses with previous studies of UK daily extremes to better understand the relative importance of different processes across timescales.

In southeast England, more than half of wet day rainfall totals in summer are typically derived from 1 h rainfall bursts, decreasing to the north and west, a pattern corresponding to that of mean maximum daily temperature (maps of UK climatological means may be accessed at the UK Met Office http://www.metoffice.gov.uk/public/weather/climate/). This suggests a possible diurnal cycle in intense hourly rainfall, which is identified in the occurrence of the *n*‐largest events in summer, showing a peak frequency in the late afternoon/early evening. This seems to occur relatively consistently across the UK, although a similar cycle detected in spring is most evident once again in eastern coastal regions. This cycle is likely driven by convection that is at a maximum at this time of day, associated with the diurnal peak of temperature, although further investigation is required into the local‐ and large‐scale drivers of intense rainfall. Extreme UK daily precipitation in winter and autumn has been shown to be strongly influenced by synoptic‐scale ‘atmospheric rivers’ (Lavers and Villarini, 2013), and these have been identified as an important mechanism in causing floods (Lavers *et al.*, 2011), but large‐scale precursors of intense summer rainfall are less well understood, even on daily timescales (Champion *et al.*, 2015). Furthermore, large‐scale drivers may also be modified by local climatic factors, for example, Svensson and Jakob (2002) identified a higher frequency of winter events of at least 5 mm h^−1^ in the early hours of the morning for a site in southern Scotland. This is associated with the orographical enhancement of precipitation after radiative night‐time cooling leads to an increase in relative humidity but is strongly linked to wind direction. Coupled with information derived from high‐resolution models, these observations may therefore be used to improve understanding of how large‐scale dynamics interact with local‐scale thermodynamic processes as drivers of intense rainfall (e.g. Blenkinsop *et al.*, 2015) and lead to a better understanding of the potential drivers of flash flooding from intense rainfall.

A further benefit gained from the assessment of these fundamental climatological characteristics of extremes is as a complement to the QC process. It is recommended therefore that relatively simple analyses should form part of the QC process for any observed precipitation dataset. This may be achieved through comparison of the characteristics of individual gauges with a regional climatology, for example, a period of suspect data was identified at a location with a significantly higher frequency of 1 h ERA events than at other gauges (or indeed higher than expected for this magnitude of event). Alternatively, such analyses might include an assessment of the climatology of gauges in relation to known processes. Two examples of the latter from this analysis include the highlighting of high‐intensity events in winter (these events were shown to be most closely associated with summer) and the identification of 0900‐accumulated totals in some rain gauges producing a corresponding morning spike in the diurnal cycle of extreme events. The seasonality and timing of extremes might therefore form part of a suite of essential QC procedures and diagnostic analyses for sub‐daily precipitation data.

Given the association between periods of intense rainfall and flash flooding, there is considerable interest in potential future changes associated with a warming climate. The dataset developed here may therefore contribute to a number of hydro‐climatological applications:

- Typically, regional climate models have been noted to have limited skill in the simulation of UK summer rainfall extremes on both daily (Fowler and Ekström, 2009) and sub‐daily (Chan *et al.*, 2014) timescales. This work provides a high‐quality dataset against which some aspects of the performance of the emerging generation of very high‐resolution, convection‐permitting climate models (e.g. Kendon *et al.*, 2012; Ban *et al.*, 2014) may be assessed. These models are also able to provide output variables that are simulated at a higher temporal resolution (e.g. 10 min), and so, gauges for which tip times are available may also provide further value in this context.
- The dataset includes a limited number of longer records that are suitable, subject to homogeneity testing, for the analysis of long‐term trends and variability.
- There is considerable demand for hourly rainfall products within the hydrological community for climate change impact assessments. Dale *et al.* (2015) used a small selection of these hourly gauges as analogues for future climate change in assessing potential uplift factors associated with intense rainfall for sewer design. There is also demand for a high‐resolution gridded hourly dataset, particularly for hydrological modelling. Consequently, the complete quality‐controlled hourly rainfall dataset is being used to construct a gridded 1 km product.
- For the UK, the UKCP09 Weather Generator (Jones *et al.*, 2009) is used to downscale regional climate model output to a 5 km grid, but hourly rainfall statistics are estimated using regression relationships between 1 h and 1 d statistics from 35 hourly gauges across the UK. The identification of spatially and temporally varying relationships between these different accumulation periods from this more extensive dataset could improve downscaled projections of future hourly rainfall.

Given these potential applications, expanding the availability of sub‐daily precipitation data globally is a key priority for the climatology community. The suite of QC procedures described here will support a global initiative to collate such datasets. This exercise is currently being undertaken by the INTENSE (INTElligent use of climate models for adaptatioN to non‐Stationary hydrological Extremes) project as a contribution to the Global Energy and Water Cycle Experiment (GEWEX) Grand Challenge on Extremes. This has identified the importance of efforts to undertake new and novel QC algorithms at different timescales (Alexander *et al.*, 2015), and the work begun here makes a contribution towards this and their stated aim of creating an integrated set of holdings of *in situ* data over global land areas, of which sub‐daily precipitation data is a key component.

## Supporting information


**Appendix S1**. Additional analysis of the QC procedures and climatology of extremes, and a more detailed account of the FEH methodology for calculating the circular statistics.

**Figure S1**. Time series of the mean proportions (p) of the three classes of quality‐control metadata for EA gauges for three regions and the number of contributing gauges (n).

**Figure S2**. Relative frequencies of data flagged by the main quality‐control procedures applied to all data.

**Figure S3**. Change in seasonal 1 h R_med_ after additional quality‐control procedures.

**Figure S4**. Number of recorded 1 h extreme rainfall alert (ERA) threshold events (≥30 mm h^−1^) removed by additional quality‐control procedures.

**Figure S5**. Seasonal mean wet hour intensity.

**Figure S6**. Seasonal 99th percentile wet hour amount.

**Figure S7**. Seasonal 99.9th percentile wet hour amount.

**Figure S8**. Frequency of 1 h extreme rainfall alert (ERA) threshold events (≥30 mm h^−1^) after additional quality‐control procedures.

**Figure S9**. Timing and magnitude of n1 events by extreme rainfall region.

## References

[joc4735-bib-0001] Alexander L , Zhang X , Hegerl G , Seneviratne S . 2015 *Implementation plan for WCRP grand challenge on understanding and predicting weather and climate extremes* World Climate Research Programme Report: Geneva, Switzerland. http://www.wcrp‐climate.org/gc‐extremes‐documents (accessed 16 June 2015).

[joc4735-bib-0002] Allen MR , Ingram WJ . 2002 Constraints on future changes in climate and the hydrologic cycle. Nature 419: 224–232. 12226677 10.1038/nature01092

[joc4735-bib-0003] Archer DR , Fowler HJ . 2015 Characterising flash flood response to intense rainfall and impacts using historical information and gauged data in Britain. J. Flood Risk Manage, doi: 10.1111/jfr3.12187.

[joc4735-bib-0004] Ban N , Schmidli J , Schär C . 2014 Evaluation of the convection‐resolving regional climate modeling approach in decade‐long simulations. J. Geophys. Res. 119: 7889–7907.

[joc4735-bib-0005] Berg P , Moseley C , Haerter JO . 2013 Strong increase in convective precipitation in response to higher temperatures. Nat. Geosci. 6: 181–185.

[joc4735-bib-0006] Blenkinsop S , Chan SC , Kendon EJ , Roberts NM , Fowler HJ . 2015 Temperature influences on intense UK hourly precipitation and dependency on large‐scale circulation. Environ. Res. Lett. 10: 054021.

[joc4735-bib-0007] Champion AJ , Allan RP , Lavers DA . 2015 Atmospheric rivers do not explain UK summer extreme rainfall. J. Geophys. Res. Atmos. 120: 6731–6741, doi: 10.1002/2014JD022863.

[joc4735-bib-0008] Chan SC , Kendon EJ , Fowler HJ , Blenkinsop S , Roberts N , Ferro CAT . 2014 The value of high‐resolution Met Office regional climate models in the simulation of multi‐hourly precipitation extremes. J. Clim. 27: 6155–6174.

[joc4735-bib-0010] Dale M , Luck B , Fowler HJ , Blenkinsop S , Gill E , Bennett J , Kendon E , Chan S . 2015 New climate change rainfall estimates for sustainable drainage. Proceedings of the Institution of Civil Engineers. Engineering Sustainability. doi: 10.1680/jensu.15.00030.

[joc4735-bib-0011] Defra . 2012. The UK climate change risk assessment 2012 evidence report, Defra: London, UK.

[joc4735-bib-0012] van Delden A . 2001 The synoptic setting of thunderstorms in western Europe. Atmos. Res. 56: 89–110.

[joc4735-bib-0013] Deshpande NR , Kulkarni A , Kumar K . 2012 Characteristic features of hourly rainfall in India. Int. J. Climatol. 32: 1730–1744.

[joc4735-bib-0014] Doe RK . 2004 Extreme precipitation and run‐off induced flash flooding at Boscastle, Cornwall, UK – 16 August 2004. J. Meteorol. 29: 319–333.

[joc4735-bib-0015] Dunn RJH , Willett KM , Thorne PW , Woolley EV , Durre I , Dai A , Parker DE , Vose RS . 2012 HadISD: a quality‐controlled global synoptic report database for selected variables at long‐term stations from 1973–2011. Clim. Past 8: 1649–1679.

[joc4735-bib-0016] Eden P . 2008 Great British Weather Disasters. Continuum: London, 351 pp.

[joc4735-bib-0017] Faulkner D . 1999 Flood Estimation Handbook. 2: Rainfall Frequency Estimation. Institute of Hydrology: Wallingford, UK.

[joc4735-bib-0018] Faulkner DS , Prudhomme C . 1998 Mapping an index of extreme rainfall across the UK. Hydrol. Earth Syst. Sci. 2: 183–194.

[joc4735-bib-0019] Ferro CAT , Segers J . 2003 Inference for clusters of extreme values. J. R. Stat. Soc. Ser. B (Stat. Methodol.) 65: 545–556.

[joc4735-bib-0020] Fowler HJ , Ekström M . 2009 Multi‐model ensemble estimates of climate change impacts on UK seasonal precipitation extremes. Int. J. Climatol. 29: 385–416.

[joc4735-bib-0021] Fowler HJ , Kilsby CG . 2003a A regional frequency analysis of United Kingdom extreme rainfall from 1961 to 2000. Int. J. Climatol. 23: 1313–1334.

[joc4735-bib-0022] Fowler HJ , Kilsby CG . 2003b Implications of changes in seasonal and annual extreme rainfall. Geophys. Res. Lett. 30: 1720.

[joc4735-bib-0023] Fujibe F , Yamazaki N , Katsuyama M , Kobayashi K . 2005 The increasing trend of intense precipitation in japan based on four‐hourly data for a hundred years. SOLA 1: 41–44.

[joc4735-bib-0024] Halcrow . 2008 Proposed pluvial flooding trial service. *Report prepared for the Meteorological Office*. Halcrow: UK.

[joc4735-bib-0025] Hand W , Fox N , Collier C . 2004 A study of the twentieth‐century extreme rainfall events in the United Kingdom with implications for forecasting. Meteorol. Appl. 11: 15–31.

[joc4735-bib-0026] Hardwick Jones R , Westra S , Sharma A . 2010 Observed relationships between extreme sub‐daily precipitation, surface temperature, and relative humidity. Geophys. Res. Lett. 37: L22805.

[joc4735-bib-0027] Holley DM , Dorling SR , Steele CJ , Earl N . 2014 A climatology of convective available potential energy in Great Britain. Int. J. Climatol. 34: 3811–3824.

[joc4735-bib-0028] Holt MA , Hardaker PJ , McLelland GP . 2001 A lightning climatology for Europe and the UK, 1990–99. Weather 56: 290–296.

[joc4735-bib-0029] Hurford AP , Priest SJ , Parker DJ , Lumbroso DM . 2012a The effectiveness of extreme rainfall alerts in predicting surface water flooding in England and Wales. Int. J. Climatol. 32: 1768–1774.

[joc4735-bib-0030] Hurford AP , Parker DJ , Priest SJ , Lumbroso DM . 2012b Validating the return period of rainfall thresholds used for Extreme Rainfall Alerts by linking rainfall intensities with observed surface water flood events. J. Flood Risk Manage. 5: 134–142.

[joc4735-bib-0031] Jakob D , Karoly DJ , Seed A . 2011a Non‐stationarity in daily and sub‐daily intense rainfall – part 1: Sydney, Australia. Nat. Hazards Earth Syst. Sci. 11: 2263–2271.

[joc4735-bib-0032] Jakob D , Karoly DJ , Seed A . 2011b Non‐stationarity in daily and sub‐daily intense rainfall – Part 2: regional assessment for sites in south‐east Australia. Nat. Hazards Earth Syst. Sci. 11: 2273–2284.

[joc4735-bib-0033] Jenkins GJ , Perry MC , Prior MJ . 2008 The Climate of the United Kingdom and Recent Trends. Met Office Hadley Centre: Exeter, UK.

[joc4735-bib-0034] Jones PD , Kilsby CG , Harpham C , Glenis V , Burton A . 2009. UK climate projections science report: projections of future daily climate for the UK from the weather generator. University of Newcastle, Newcastle, UK.

[joc4735-bib-0035] Jones MR , Fowler HJ , Kilsby CG , Blenkinsop S . 2013 An assessment of changes in seasonal and annual extreme rainfall in the UK between 1961 and 2009. Int. J. Climatol. 33: 1178–1194.

[joc4735-bib-0036] Jones MR , Blenkinsop S , Fowler HJ , Kilsby CG . 2014 Objective classification of extreme rainfall regions for the UK and updated estimates of trends in regional extreme rainfall. Int. J. Climatol. 34: 751–765.

[joc4735-bib-0037] Kendon EJ , Roberts NM , Senior CA , Roberts MJ . 2012 Realism of rainfall in a very high resolution regional climate model. J. Clim. 25: 5791–5806.

[joc4735-bib-0038] Lavers DA , Villarini G . 2013 The nexus between atmospheric rivers and extreme precipitation across Europe. Geophys. Res. Lett. 40: 3259–3264.

[joc4735-bib-0039] Lavers DA , Allan RP , Wood EF , Villarini G , Brayshaw DJ , Wade AJ . 2011 Winter floods in Britain are connected to atmospheric rivers. Geophys. Res. Lett. 38: L23803.

[joc4735-bib-0040] Leahy P , Kiely G . 2011 Short duration rainfall extremes in Ireland: influence of climatic variability. Water Resour. Manag. 25: 987–1003.

[joc4735-bib-0041] Lenderink G , van Meijgaard E . 2008 Increase in hourly precipitation extremes beyond expectations from temperature changes. Nat. Geosci. 1: 511–514.

[joc4735-bib-0042] Lenderink G , Mok HY , Lee TC , van Oldenborgh GJ . 2011 Scaling and trends of hourly precipitation extremes in two different climate zones – Hong Kong and the Netherlands. Hydrol. Earth Syst. Sci. 15: 3033–3041.

[joc4735-bib-0043] Maraun D , Osborn TJ , Gillett NP . 2008 United Kingdom daily precipitation intensity: improved early data, error estimates and an update from 2000 to 2006. Int. J. Climatol. 28: 833–842.

[joc4735-bib-0044] Maraun D , Rust HW , Osborn TJ . 2009 The annual cycle of heavy precipitation across the United Kingdom: a model based on extreme value statistics. Int. J. Climatol. 29: 1731–1744.

[joc4735-bib-0045] Met Office . 2012 *Met Office Integrated Data Archive System (MIDAS) land and marine surface stations data (1853‐current)* NCAS British Atmospheric Data Centre. http://catalogue.ceda.ac.uk/uuid/220a65615218d5c9cc9e4785a3234bd0 (accessed 14 June 2015).

[joc4735-bib-0046] Muschinski T , Katz JI . 2013 Trends in hourly rainfall statistics in the United States under a warming climate. Nat. Clim. Change 3: 577–580.

[joc4735-bib-0009] Newcastle City Council . 2013. Summer 2012 flooding in Newcastle Upon Tyne. Total Research and Technical Services Report. Newcastle City Council.

[joc4735-bib-0047] Osborn TJ , Hulme M . 2002 Evidence for trends in heavy rainfall events over the UK. Philos. Trans. A Math. Phys. Eng. Sci. 360: 1313–1325. 12809141 10.1098/rsta.2002.1002

[joc4735-bib-0048] Osborn TJ , Hulme M , Jones PD , Basnett TA . 2000 Observed trends in the daily intensity of United Kingdom precipitation. Int. J. Climatol. 20: 347–364.

[joc4735-bib-0049] Pall P , Allen MR , Stone DA . 2007 Testing the Clausius–Clapeyron constraint on changes in extreme precipitation under CO_2_ warming. Clim. Dyn. 28: 351–363.

[joc4735-bib-0050] Perry MC , Hollis D , Elms M . 2009. The generation of daily gridded datasets of temperature and rainfall for the UK. Met Office National Climate Information Centre Climate Memorandum No. 24, Met Office: Exeter, UK.

[joc4735-bib-0051] Reed DW , Faulkner DS , Stewart EJ . 1999 The FORGEX method of rainfall growth estimation II: description. Hydrol. Earth Syst. Sci. 3: 197–203.

[joc4735-bib-0052] Ribatet M. 2012 *POT: generalized Pareto distribution and peaks over threshold. R package version 1.1‐3* https://cran.r‐project.org/src/contrib/Archive/POT/ (accessed 21 June 2012).

[joc4735-bib-0053] Robson A , Reed D . 1999 Flood Estimation Handbook. 3: Statistical Procedures for Flood Frequency Estimation. Institute of Hydrology: Wallingford, UK.

[joc4735-bib-0054] Rodda HJE , Little MA , Wood RG , MacDougall N , McSharry PE . 2009 A digital archive of extreme rainfalls in the British Isles from 1866 to 1968 based on British Rainfall. Weather 64: 71–75.

[joc4735-bib-0055] Rust HW , Maraun D , Osborn TJ . 2009 Modelling seasonality in extreme rainfall: a UK case study. Eur. Phys. J. Spec. Top. 174: 99–111.

[joc4735-bib-0056] Sen Roy S . 2009 A spatial analysis of extreme hourly precipitation patterns in India. Int. J. Climatol. 29: 345–355.

[joc4735-bib-0057] Sen Roy S , Rouault M . 2013 Spatial patterns of seasonal scale trends in extreme hourly precipitation in South Africa. Appl. Geogr. 39: 151–157.

[joc4735-bib-0058] Simpson IR , Jones PD . 2014 Analysis of UK precipitation extremes derived from Met Office gridded data. Int. J. Climatol. 34: 2438–2449.

[joc4735-bib-0059] Smith L , Liang Q , James P , Lin W . 2015 Assessing the utility of social media as a data source for flood risk management using a real‐time modelling framework. J. Flood Risk Manage, doi: 10.1111/jfr3.12154.

[joc4735-bib-0060] Stewart EJ , Reed DW , Faulkner DS , Reynard NS . 1999 The FORGEX methid of rainfall growth estimation I: review of requirement. Hydrol. Earth Syst. Sci. 3: 187–195.

[joc4735-bib-0061] Svensson C , Jakob D . 2002 Diurnal and seasonal characteristics of precipitation at an upland site in Scotland. Int. J. Climatol. 22: 587–598.

[joc4735-bib-0062] Trenberth KE , Dai A , Rasmussen RM , Parsons DB . 2003 The changing character of precipitation. Bull. Am. Meteorol. Soc. 84: 1205–1217.

[joc4735-bib-0063] Upton GJG , Rahimi AR . 2003 On‐line detection of errors in tipping‐bucket raingauges. J. Hydrol. 278: 197–212.

[joc4735-bib-0064] Westra S , Sisson SA . 2011 Detection of non‐stationarity in precipitation extremes using a max‐stable process model. J. Hydrol. 406: 119–128.

[joc4735-bib-0065] Westra S , Evans JP , Mehrotra R , Sharma A . 2013 A conditional disaggregation algorithm for generating fine time‐scale rainfall data in a warmer climate. J. Hydrol. 479: 86–99.

[joc4735-bib-0066] Westra S , Fowler HJ , Evans JP , Alexander LV , Berg P , Johnson F , Kendon EJ , Lenderink G , Roberts NM . 2014 Future changes to the intensity and frequency of short‐duration extreme rainfall. Rev. Geophys. 52: 522–555.

[joc4735-bib-0067] Wilks DS . 2006 Statistical Methods in the Atmospheric Sciences, 2nd edn. Academic Press: San Diego, CA.

[joc4735-bib-0068] WRc . 2006 Sewers for Adoption – Design and Construction Guide for Developers, 6th edn. WRc: Swindon, UK.

