# Supplementary material for: Quality‐control of an hourly rainfall dataset and climatology of extremes for the UK
Source: Int J Climatol. 2016 Apr 24;37(2):722–40. doi: 10.1002/joc.4735 (PMC5300158; doi:10.1002/joc.4735)
Supplement: Supplementary file 1 — Appendix S1. Additional analysis of the QC procedures and climatology of extremes, and a more detailed account of the FEH methodology for calculating the circular statistics. [file JOC-37-722-s001.pdf]

Quality control of an hourly rainfall dataset and climatology of extremes for the UK

S. Blenkinsop[1], E Lewis[1], S.C. Chan[1,2], H.J. Fowler[1]

[1]Water Resource Systems Research Laboratory, School of Civil Engineering and Geosciences, Newcastle University, Newcastle upon Tyne, NE1 7RU, UK

[2]Visiting scientist at the Met Office Hadley Centre, Exeter, UK.

SUPPORTING INFORMATION

Data, quality control and methodology

The main text of this paper identified potential data quality problems associated with sub-daily rainfall data from three sources in the UK and outlined procedures for detecting these in rainfall time series. Here we provide quantitative estimates of the extent to which the data are affected by these issues and their potential effect on some measures of extremes.

Assessment of pre-existing data quality control

An assessment of the pre-existing Environment Agency (EA) quality control (QC) metadata (identifying data as good, suspect or unchecked with reference to a check gauge) indicated that most data is identified as good, although a sufficient proportion of the data is unchecked so as to necessitate further quality control procedures. Table S1 provides a seasonal breakdown of the data quality metadata and indicates that although the proportion of data identified as good is reasonably consistent at different times of the year there is some evidence that less data is checked in summer (a gauge average of ~30% unchecked compared with ~28% averaged across the other seasons). The highest proportion of suspect data occurs in winter which may in part be a consequence of problems with snow and frozen gauges. Such instances were recorded in free-format fields in the metadata for some gauges but this information is not consistently recorded across the gauge network.

There is however considerable spatial variability across the UK, suggesting that observation and validation practice across the EA's administrative regions are not standardised. The average proportion of unchecked data per raingauge ranges from ~1.5% to ~61% across 19 different administrative sub-regions, whilst suspect data ranges from ~0% to ~19%. The tendency for more data to be unchecked in summer also varies across the country but is particularly evident in south-east England where the summer-winter differential in some areas is ~+10%. The lowest proportions of suspect data occur in south-east England with corresponding high levels of good data. Such differences may arise as a consequence of a better maintained, more reliable gauge network in some regions but conversely could also arise due to less stringent use of check gauges and field inspections. Without a detailed knowledge of operational procedures it is not possible to determine to which of these causes the differences may be attributed.

	**Good (%)**	**Suspect (%)**	**Unchecked (%)**
**Annual**	62.5	9.3	28.2
**DJF**	61.9	10.9	27.2
**MAM**	63.0	8.4	28.6
**JJA**	61.2	9.1	29.7
**SON**	64.2	8.6	27.2

Table S1: Percentage distribution of EA quality control metadata from check gauges. Data is presented for metadata associated with all non-zero rainfall amounts. Where the nature of the data quality is unclear i.e. the metadata indicates the rainfall amount has been estimated or manually edited this has been categorized as "unchecked" for the purposes of this analysis.

Regional differences are also apparent in the temporal evolution of data quality. The main paper identified a large increase in unchecked data centred at around the mid-2000s. A more detailed exploration of this data shows that this pattern is apparent in many of the EA's administrative regions, as illustrated by the case of North Wales in Figure S1. However, raingauges in the Midlands region (shown for the East Midlands) display a pattern of gradually increasing suspect data whilst others show a combination of these two patterns. Raingauges in SW England, which was instrumented later than the rest of the country, display a completely different pattern. It is not

possible however to identify the reasons for these changes – the increase in the proportion of suspect data for example could arise as a consequence of improved checking and identification of erroneous data or, alternatively, could be due to a deterioration in the quality of the raingauges.

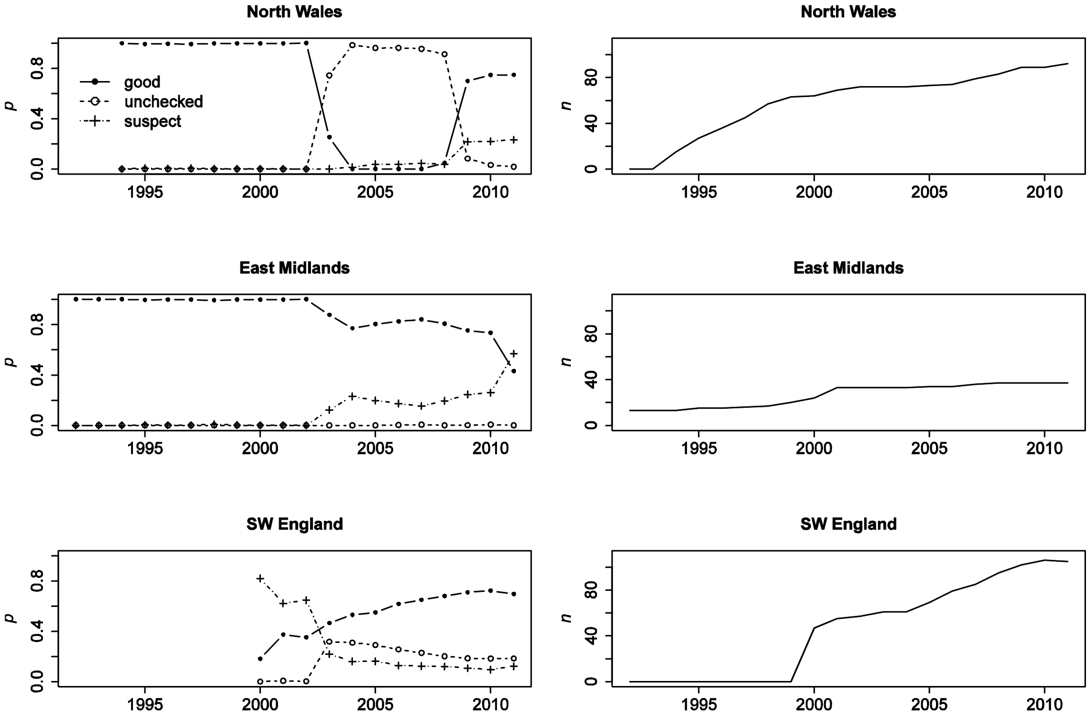

Figure S1: Time series of the mean proportions (*p*) of the three classes of quality control metadata for EA raingauges for three regions (left column) and the number of contributing raingauges (*n*) (right column).

Assessment of additional quality control procedures

Due to the spatially and temporally varying approaches to data quality assessment and clearly identifiable suspect data across all data sources that were noted in the main paper, additional QC procedures were required before any analysis of the data was undertaken. The frequencies of data flagged for each of the main procedures identified in the paper are shown in Figure S2 (each procedure (QC1-QC11) is summarized in Table 2). This shows that relatively few suspect threshold exceedances (QC1, QC2 and QC3) are identified in the Met Office Integrated Data Archive System

(MIDAS) data reflecting the quality control measures that have been undertaken before making this data available to the research community. Only one value exceeding the Met Office hourly record (QC1) was identified at Winterbourne, Birmingham (MIDAS gauge ID 586) in December 2003, with two further values > 80mm h^{-1} in the space of 4 days which were flagged by QC2. Inspection of two nearby gauges (2.5km and 13.4km away) indicated a period that was mainly dry with short, light showers, and no significant rainfall was identified in this area in the Royal Meteorological Society's *Weather Log* and so these values were considered erroneous. These problems are more common in the other two datasets where less rigorous testing has been undertaken previously and are often associated with potential daily accumulations (QC4 and QC5). These were particularly identified in the Scottish Environmental Protection Agency (SEPA) data which included a number of confirmed daily and monthly (QC6) totals. This was borne out by manual inspection of the data which indicated that for many raingauges, although hourly data was provided, they were in fact at daily resolution, with non-zero data only provided at 0900, often in the earlier part of the record. Where only daily/monthly data was included such periods were not appropriate for this dataset and analysis. Again, few problems with potential accumulated values were identified in the MIDAS raingauges though some of these flagged values did identify outstanding quality issues. For example, significant problems were associated with the raingauge at Pembrey Sands in South Wales (MIDAS gauge ID 1226), for which 16 1h Extreme Rainfall Alert (ERA) events (≥30mm) were identified over the period January to May 1993. Such a high occurrence rate is not plausible given both the expected frequency of such events (they were designed to approximate a 1 in 30 year event) and their more likely occurrence through summer convection. Further investigation indicated that the site opened on January 1st 1993 and that the high frequency of events was likely related to initial raingauge setup problems. Consequently it was determined that this period should not be included in the analysis and potentially deleted from the MIDAS database (UK Met Office, personal communication). However, not all of these erroneous hourly totals were flagged by the QC procedures described here and so would not be removed by their automated implementation. The raingauge was though highlighted for investigation by a) the incorporation of basic climatological analyses as part of the QC process, in this case examination of the consistency of the observed frequency of ERA events with expected frequencies and, b) comparison with daily rainfall products.

Suspicious duplication of values in consecutive hours (QC7) was identified as a potential problem, mainly in the EA dataset whilst frequent tipping (QC8) could only be identified in this dataset as it is the only one for which instantaneous tipping bucket tip times were available. Overall, it can be seen that the QC procedures can provide improvements to the EA data of which a significant proportion was previously unchecked. The SEPA data is most significantly affected by the provision of accumulated daily values whilst a small number of isolated problems in the MIDAS data have also been identified.

An examination of potential instances of unrecorded gauge non-operation indicated once again that the MIDAS data are least affected by ‘terminal’ dry spells (i.e. strings of zero values at the start or end of the raingauge record, QC9) with ~0.02% of gauges having such spells of at least one month duration (Figure S2). In contrast, SEPA records are most affected with ~30% of raingauges characterised by such spells, often of much longer than one month duration. All other instances where no rain occurred (‘non terminal’) for periods of greater than one month (QC10 and QC11) were flagged for further investigation alongside the matching statistics described in the main paper. These were often concentrated in a number of gauges with many dry spells, suggesting that they were, in fact, in operation sporadically (a maximum of 30 were identified in a single raingauge record).

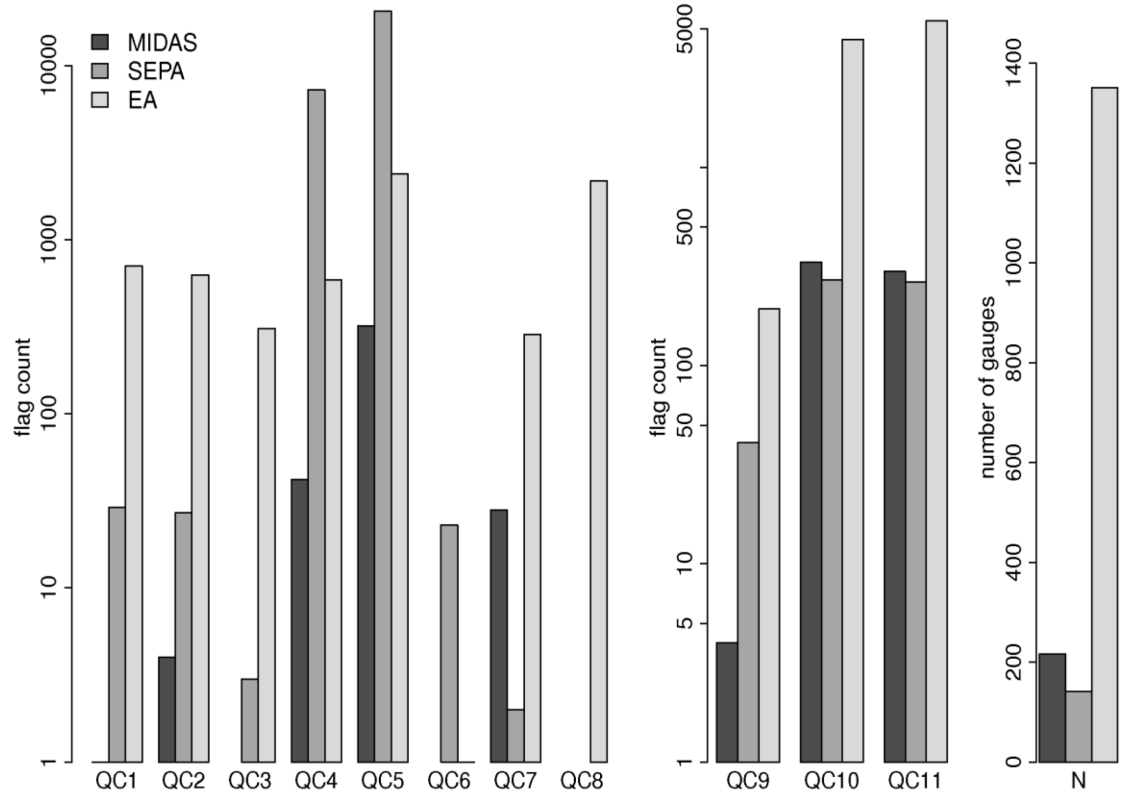

Figure S2: Frequencies of data flagged by the main quality control procedures applied to all data and described in the main text. The acronyms are defined in Table 2 in the main paper, N denotes the number of raingauges. For QC1-QC8 flag counts denote the number of hours (days for QC3) where each flag is triggered, for QC9-QC11 flag count denotes the number of identified dry spells. Note the different vertical scales and logarithmic scales in the left and centre plots.

Effect of additional quality control procedures on statistics of extreme rainfall

After all QC procedures had been completed a comparison of the resultant dataset was made with that before the additional QC had been undertaken. This comparison was undertaken for the final gauge selection using the seasonal 1h R_{med} (Figure S3) and the frequency of ERA threshold events (Figure S4). Between ~10% and ~20% of raingauges show a change in the 1h R_{med} as a consequence of the QC procedures (DJF – 10.2%, MAM – 17.6%, JJA – 18.4%, SON – 10.1%). In just over half of the cases of change (~54%) R_{med} decreases, partly due to the identification and removal of spurious

extremes, but perhaps counter-intuitively a substantial number of changes are positive (~46%). This arises due to the increased amount of missing data after the QC process which can produce a reduction in the number of years meeting the threshold for completeness and therefore potentially produce an increase in R_{med} (Figure S3). The average magnitude of the decreases in R_{med} exceeds those of the increases in all seasons and, apart from in DJF, the largest changes are negative. A total of 13 raingauges had ERA events removed by the QC process (see Figure S4 for removed events and Figure S8 for final ERA frequencies). The most notable of these were the removal of 24 consecutive hourly totals of ~40mm for two EA raingauges (flagged as consecutive duplicate values (QC7) and exceeding the 24h rainfall record (QC3)), and one SEPA gauge which had 7 events identified as accumulations recorded at 0900 (QC4 & QC5), with totals of between 39mm and 347mm. The removal of these events is also consistent with the design of ERA thresholds to approximate the average 1-in-30 year return period event.

The use of non quality controlled data affects a minority of raingauges for the statistics selected here for a broad, national scale spatial assessment of the climatology of extreme 1h rainfall (although still ~20% of calculated R_{med} values in JJA are affected). It would nonetheless potentially produce significant anomalies in assessing the seasonality and timing of extreme events. For example, its use would produce an erroneous 0900 peak in the diurnal cycle for some locations and even regions, as well as an increased number of 1h extremes in winter. Further, this would affect any subsequent analyses using extreme value statistics and regional frequency analysis to derive return period rainfall estimates.

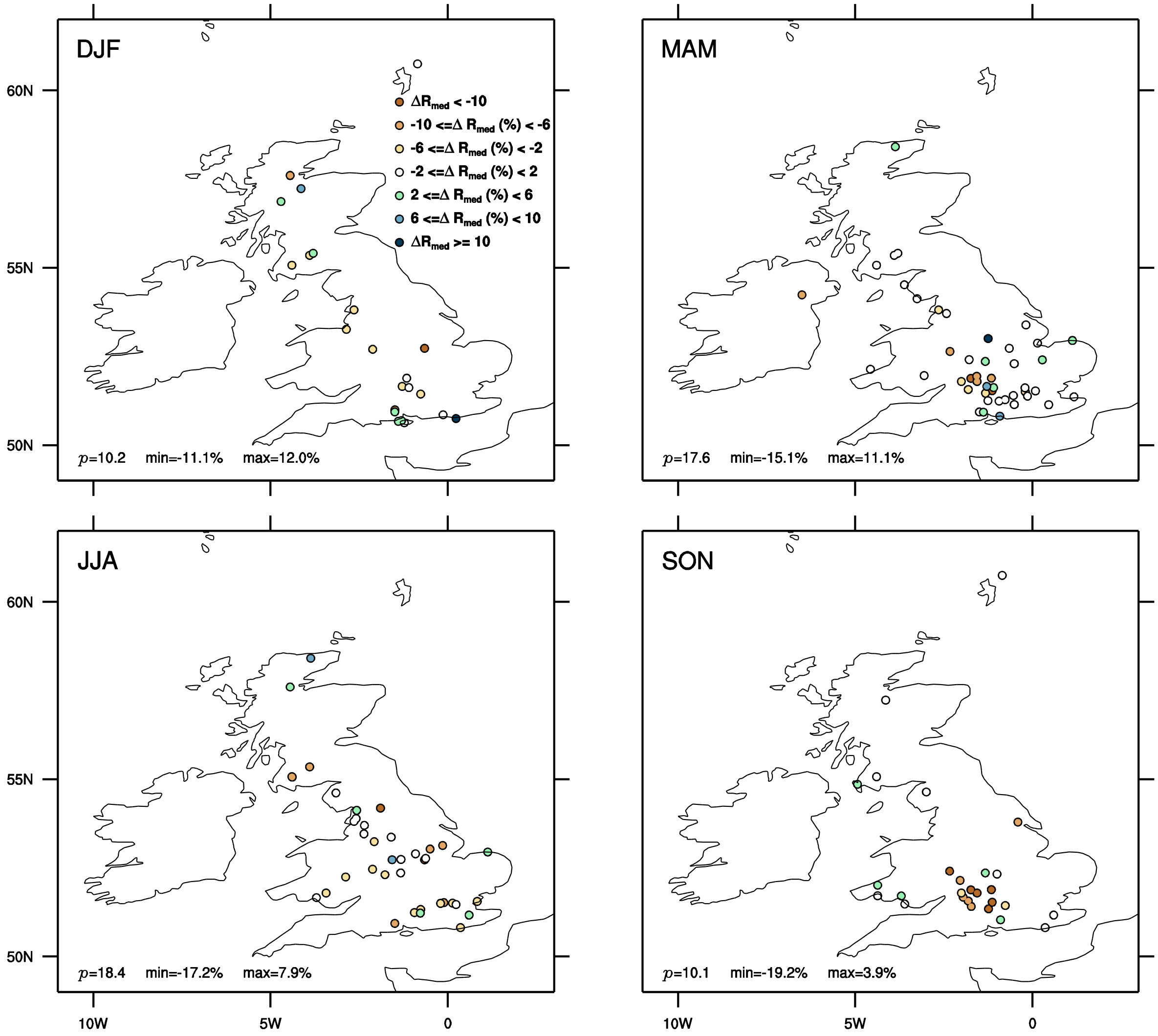

Figure S3: Change in seasonal 1h R_{med} after additional quality control procedures (expressed as a percentage of the statistic before those procedures) for the period 1992-2011, *p* denotes the proportion of gauges showing a changed statistic for each season. Max and min denote the largest positive and negative changes respectively. Only gauges showing a change are plotted for clarity.

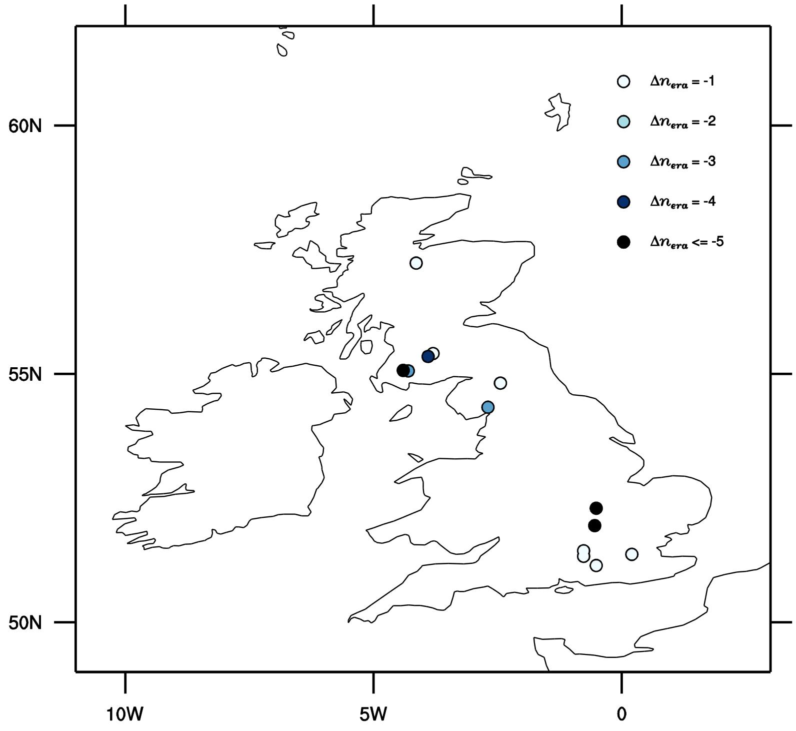

Figure S4: Number of recorded 1h extreme rainfall alert (ERA, n_{era}) threshold events (≥ 30mm h^{-1}) removed by additional quality control procedures for the period 1992-2011.

Method used to calculate seasonality statistics

Robson & Reed (1999) describe a method to estimate flood seasonality which is here applied to the *n*-largest rainfall events. Event dates are represented by the angle θ on a circle of unit radius and are calculated from the number of days since the start of the calendar year:

$$\theta = (day\ no. - 0.5)\frac{2\pi}{no.\,of\ days\ in\ year}$$

The centroid of the events may be determined by the coordinates:

$$\bar{x} = \frac{1}{n}\sum_{i=1}^{n} cos\theta_i,\ \bar{y} = \frac{1}{n}\sum_{i=1}^{n} sin\theta_i$$

The values for $\bar{r}$ and $\bar{\theta}$ may then be evaluated by:

$$\bar{\theta} = \begin{cases} tan^{-1}\left(\frac{\bar{y}}{\bar{x}}\right) & \bar{x} \geq 0, \bar{y} \geq 0 \\ tan^{-1}\left(\frac{\bar{y}}{\bar{x}}\right) + \pi & \bar{x} < 0 \\ tan^{-1}\left(\frac{\bar{y}}{\bar{x}}\right) + 2\pi & \bar{x} \geq 0, \bar{y} < 0 \end{cases}$$

$$\bar{r} = \sqrt{\bar{x}^2 + \bar{y}^2}$$

This method is applied to assess the seasonality of UK extreme hourly rainfall event in the main paper and in Figure S9.

RESULTS

Analysis of the climatology of UK hourly extremes

Additional results from the analyses of the quality controlled UK hourly rainfall are provided in Figures S5 to S9 and are referred to in the main paper. Figure S5 provides seasonal patterns of mean wet hour intensity (MWHI) whilst Figures 6-8 present patterns of percentile- and fixed-threshold (ERA) derived measures of extremes. Figure S9 presents a comprehensive regional summary of the timing and magnitude of the *n*-largest events for *n*=1.

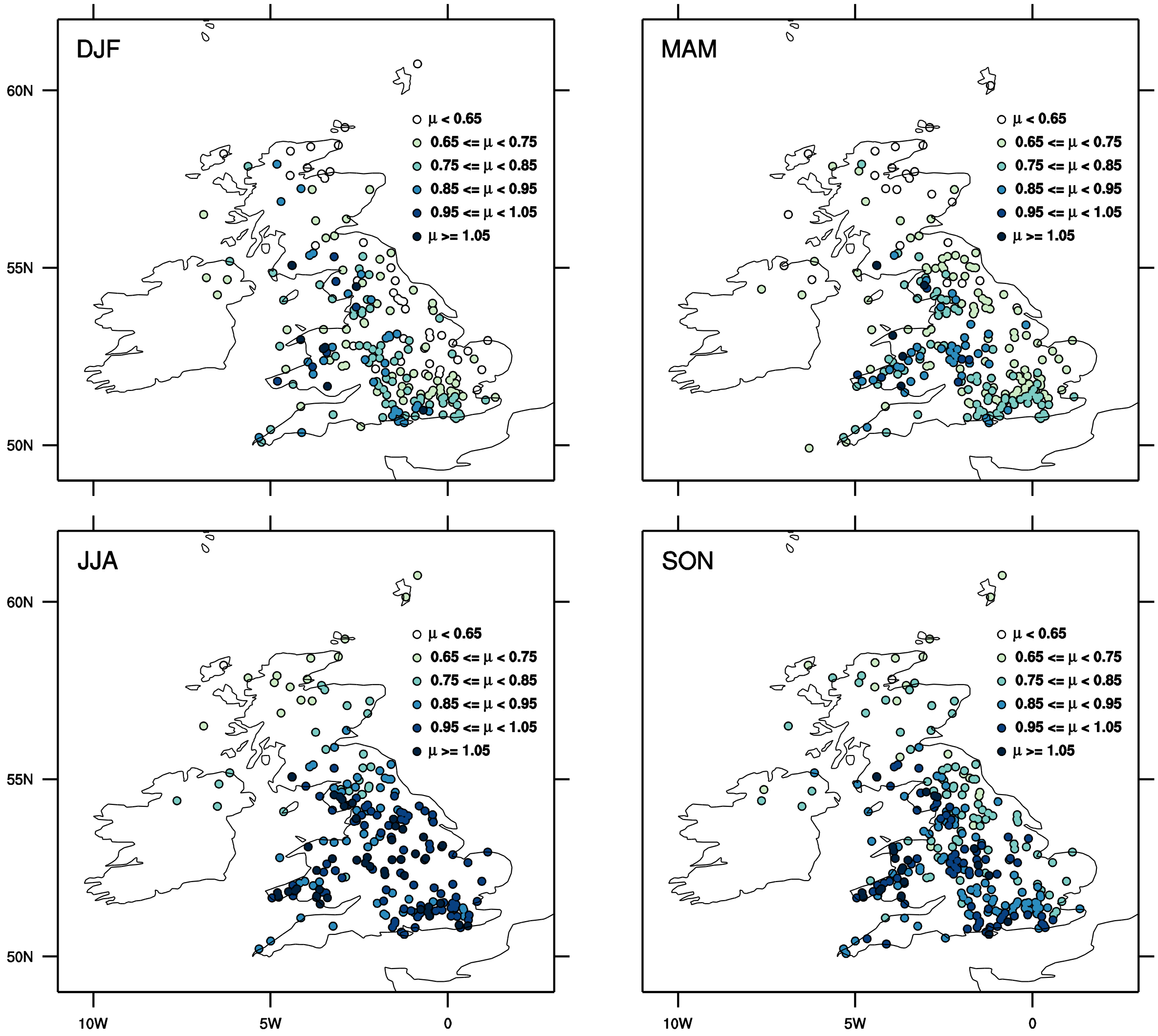

Figure S5: Seasonal mean wet hour intensity (MWHI; hours when precipitation ≥ 0.2mm), denoted by μ, for the period 1992-2011. Note different seasonal scales.

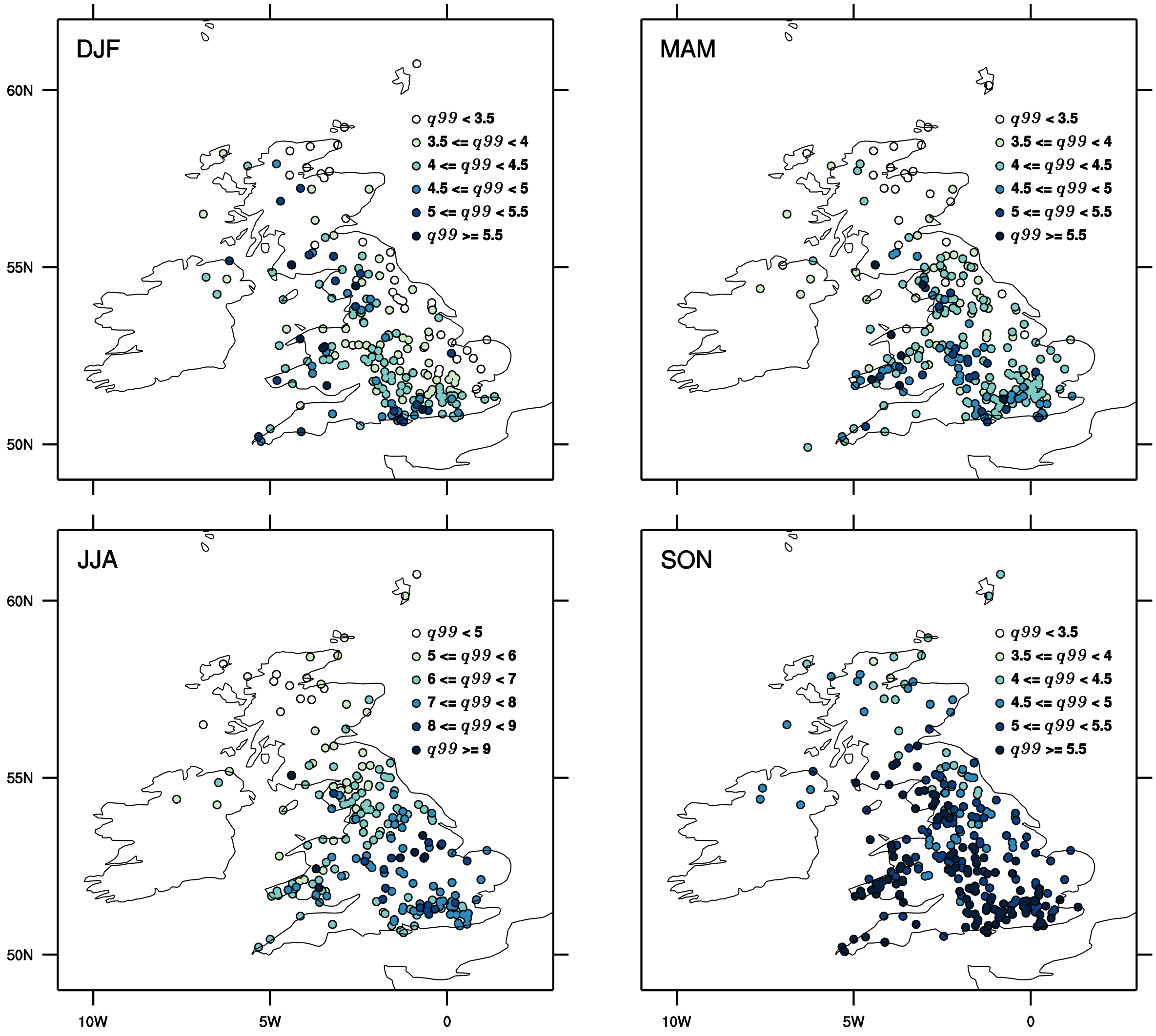

Figure S6: Seasonal 99th percentile wet hour amount (hours when precipitation ≥ 0.2mm), denoted by *q99,* for the period 1992-2011. Note different seasonal scales.

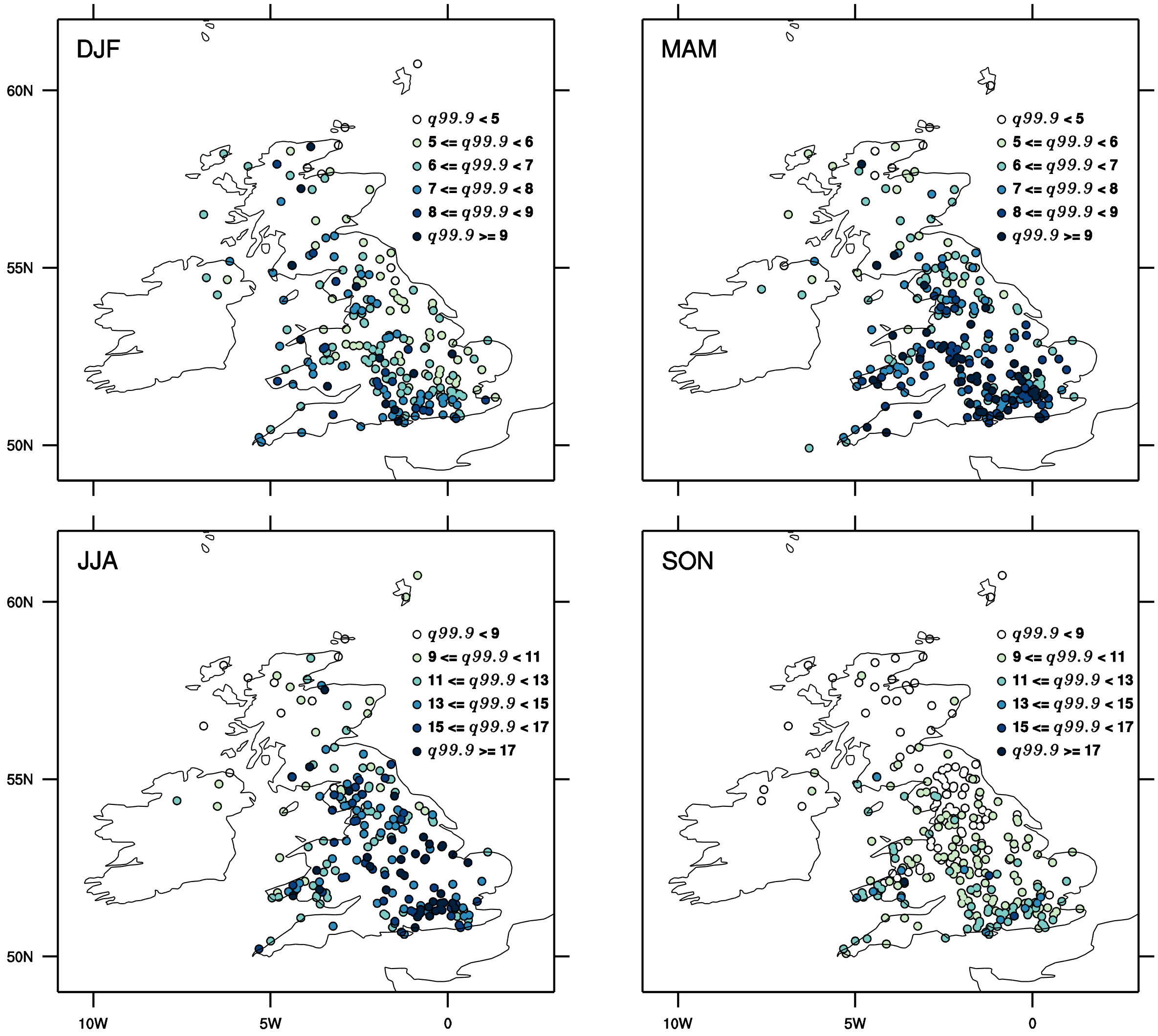

Figure S7: As for Figure S6 but for the 99.9th percentile, denoted by *q99.9*. Note different seasonal scales.

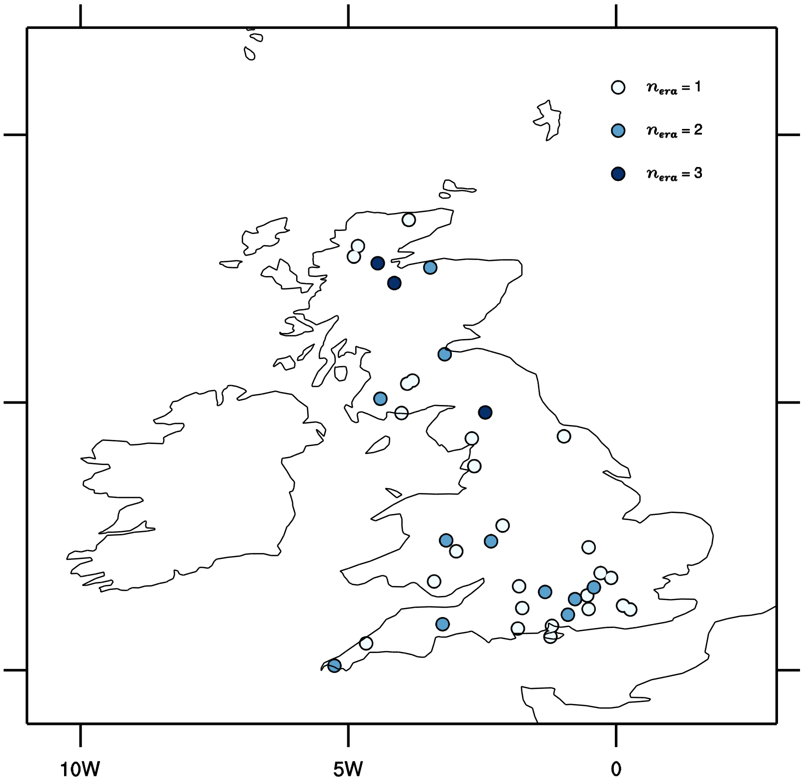

Figure S8: Frequency (n_{era}) of recorded 1h extreme rainfall alert (ERA) threshold events (≥ 30mm h^{-1}) after additional quality control procedures for the period 1992-2011.

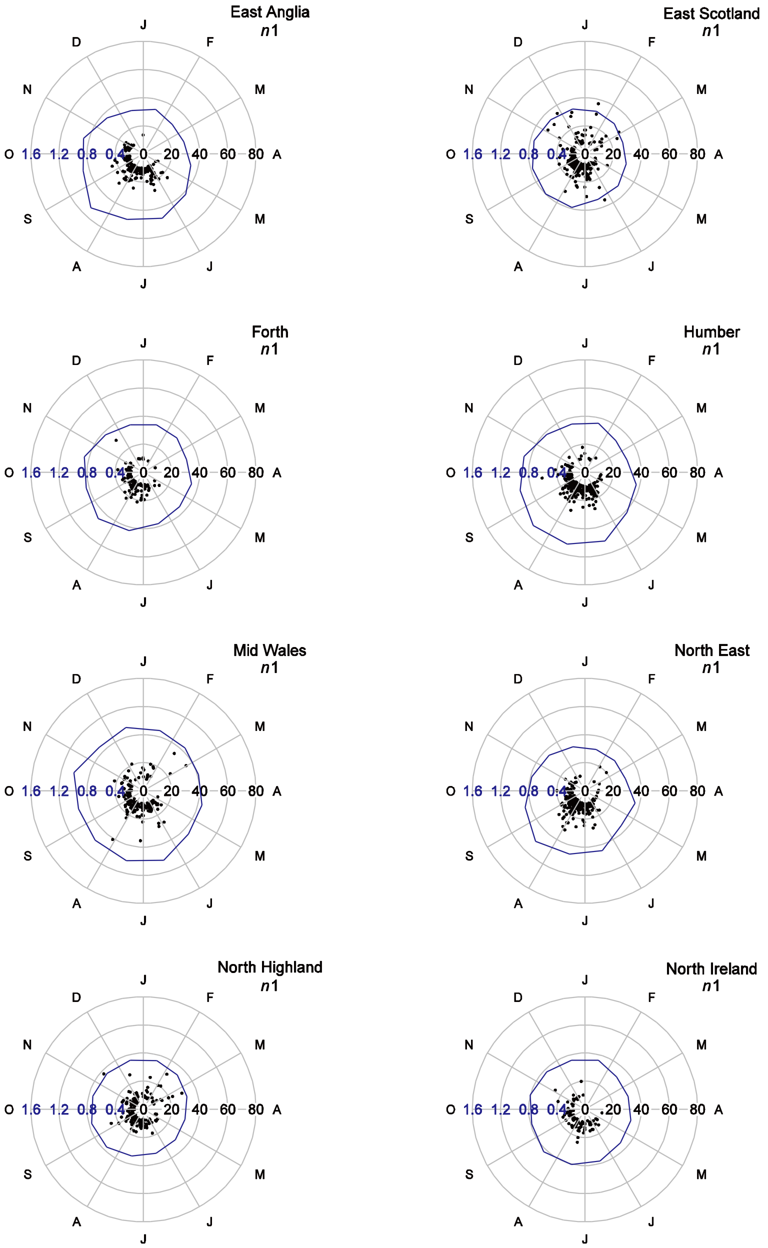

Figure S9: Timing and magnitude of n1 events (the 20 largest events for each gauge in a complete 20 year record) by extreme rainfall region. Black dots denote n-largest events with magnitude denoted by the right hand axis (in mm). Monthly mean wet hour intensity (MWHI) is shown by the solid line denoted by the left hand axis (in mm).

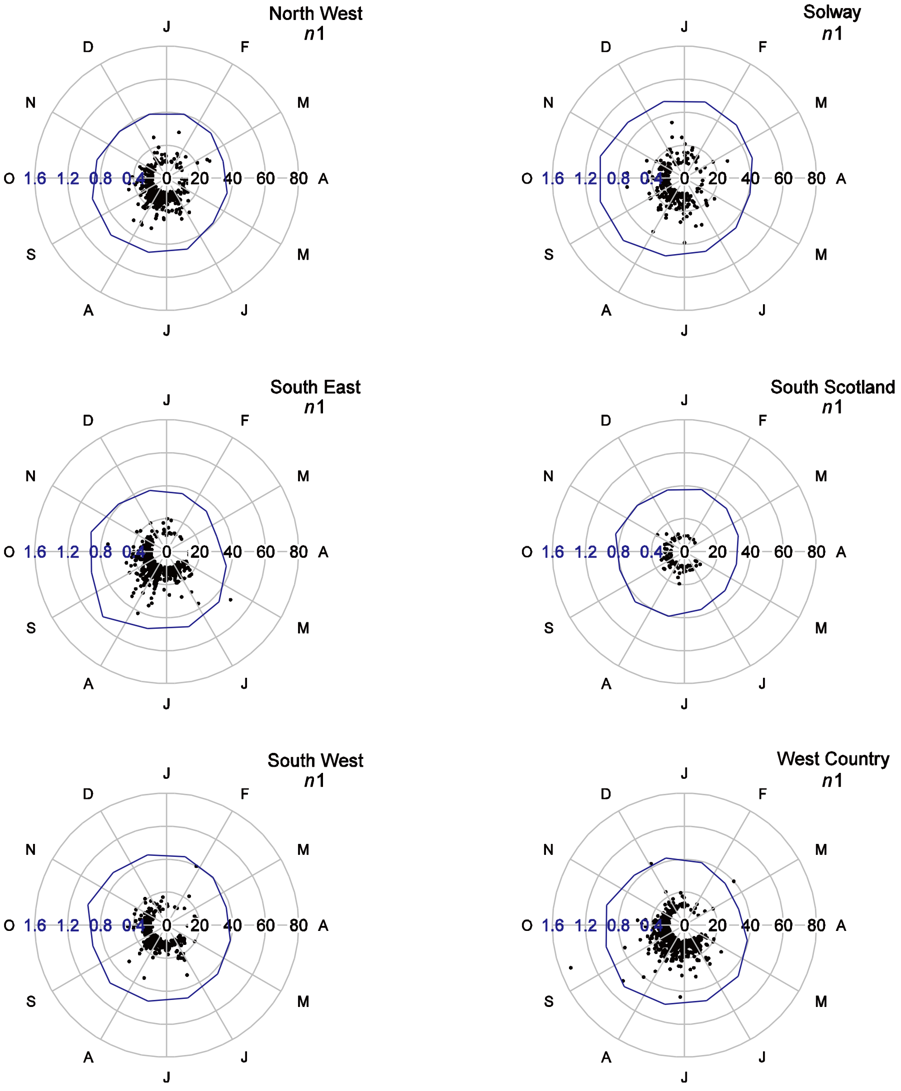

Figure S9 (continued): Timing and magnitude of *n*1 events (the 20 largest events for each gauge in a complete 20 year record) by extreme rainfall region. Black dots denote *n*-largest events with magnitude denoted by the right hand axis (in mm). Monthly mean wet hour intensity (MWHI) is shown by the solid line denoted by the left hand axis (in mm).

REFERENCES

Robson A, Reed D. 1999. *Flood Estimation Handbook. 3: Statistical procedures for flood frequency estimation*. Institute of Hydrology, Wallingford, UK.
