# Supplementary figures and images for: Quality‐control of an hourly rainfall dataset and climatology of extremes for the UK
Source: Int J Climatol. 2016 Apr 24;37(2):722–40. doi: 10.1002/joc.4735 (PMC5300158; doi:10.1002/joc.4735)

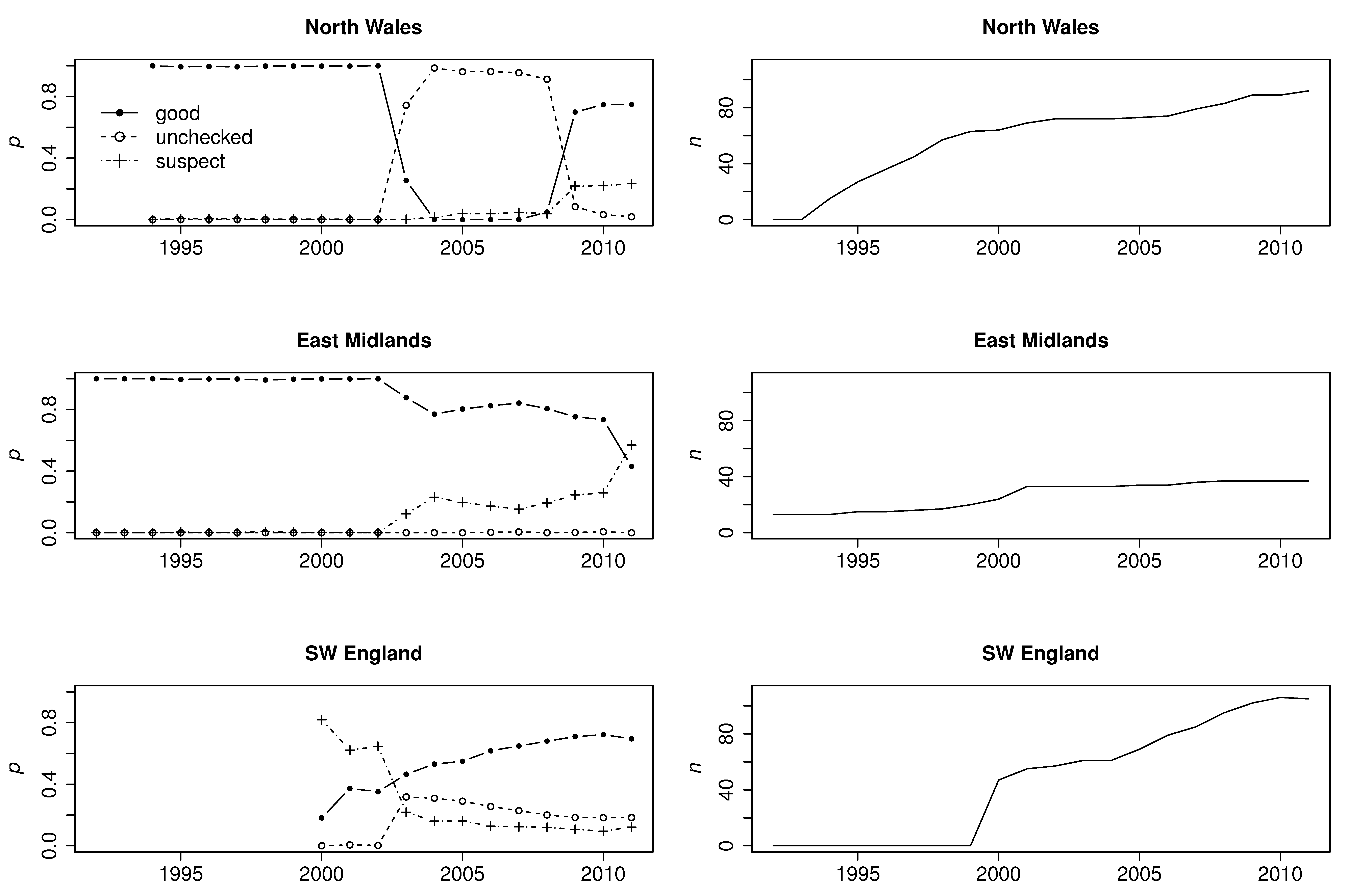

Supplement: Supplementary file 2 — Figure S1. Time series of the mean proportions (p) of the three classes of quality‐control metadata for EA gauges for three regions and the number of contributing gauges (n). [file JOC-37-722-s002.tif]

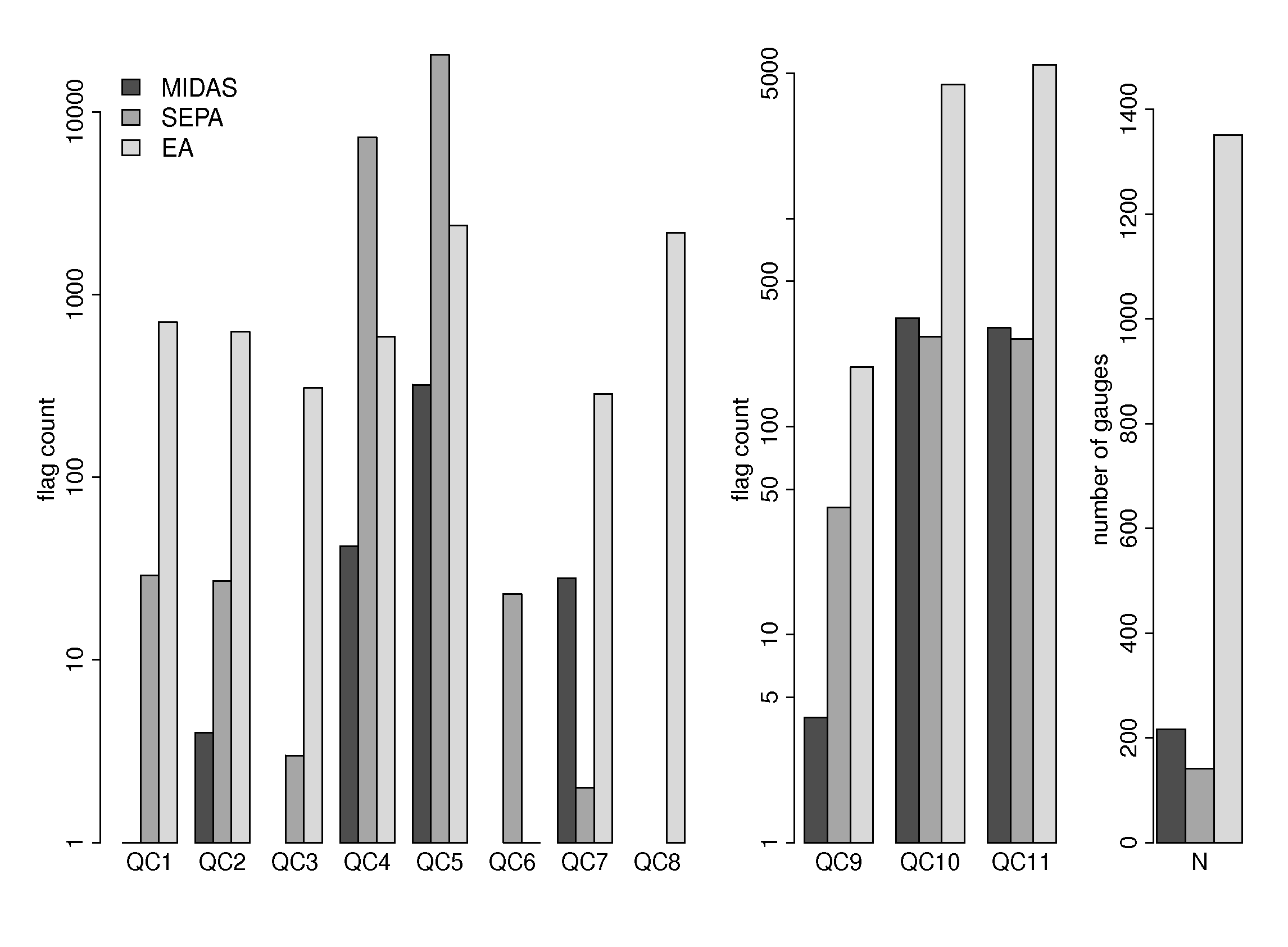

Supplement: Supplementary file 3 — Figure S2. Relative frequencies of data flagged by the main quality‐control procedures applied to all data. [file JOC-37-722-s003.tif]

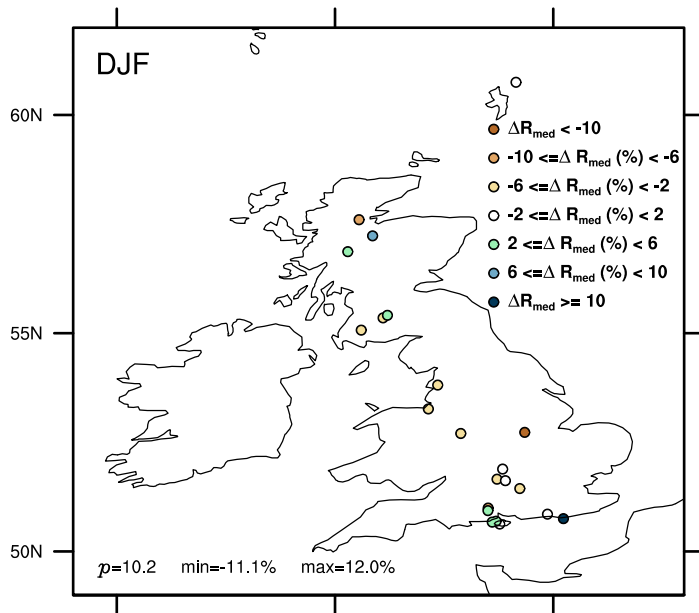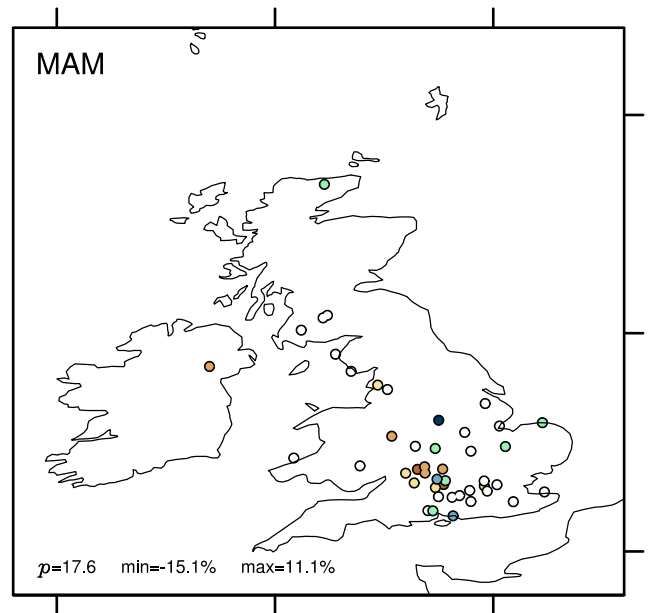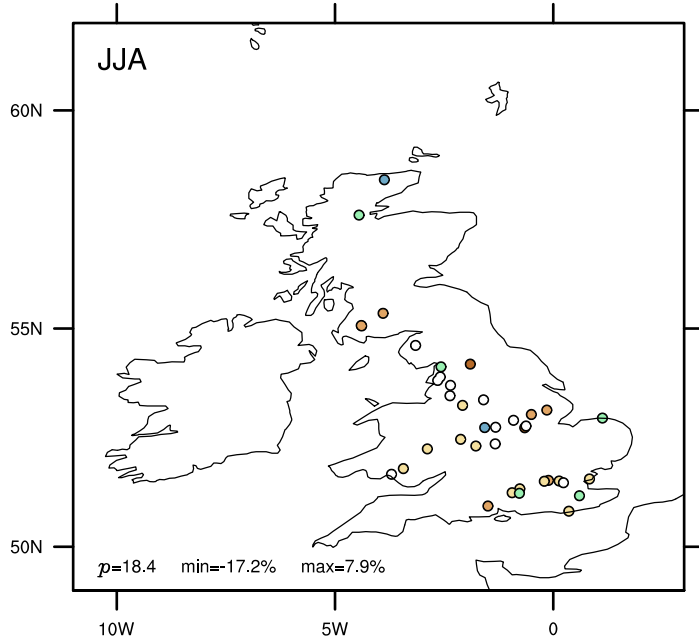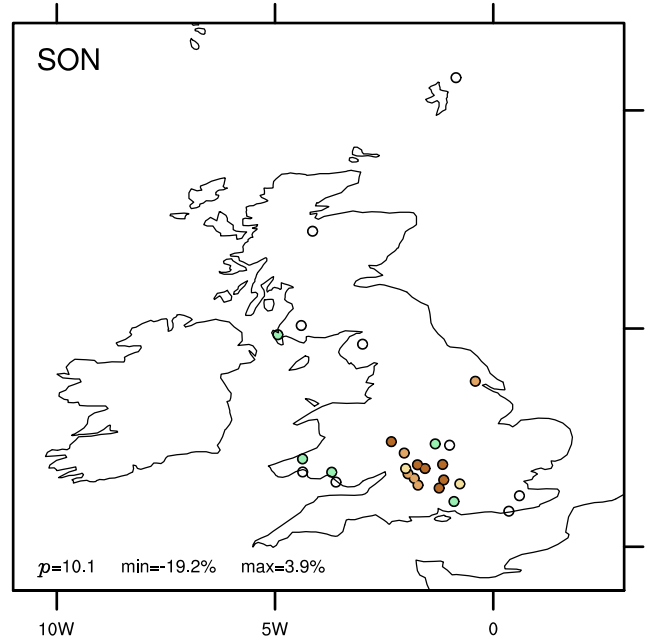

Supplement: Supplementary file 4 — Figure S3. Change in seasonal 1 h R med after additional quality‐control procedures. [file JOC-37-722-s004.pdf]

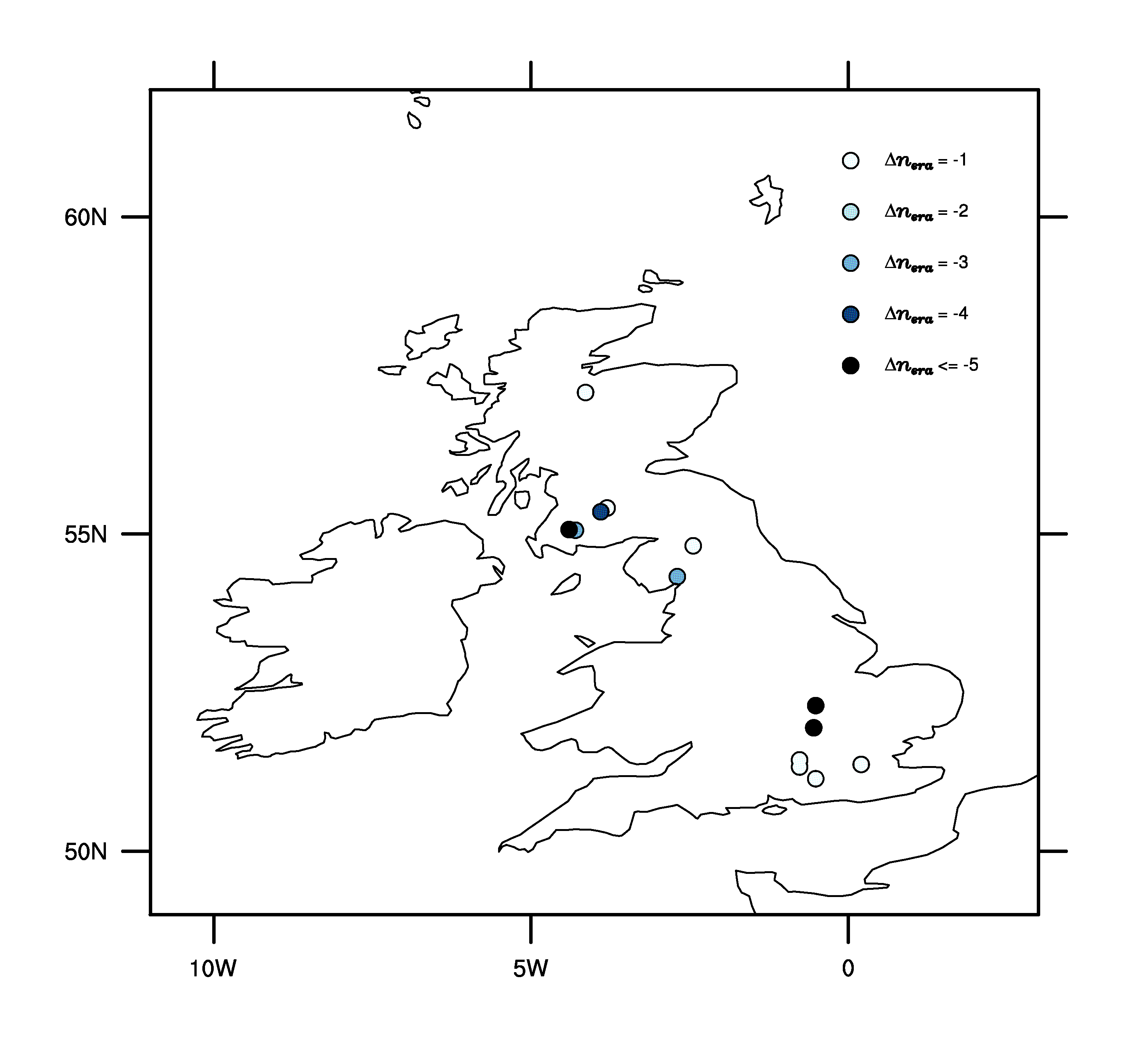

Supplement: Supplementary file 5 — Figure S4. Number of recorded 1 h extreme rainfall alert (ERA) threshold events (≥30 mm h−1) removed by additional quality‐control procedures. [file JOC-37-722-s005.tif]

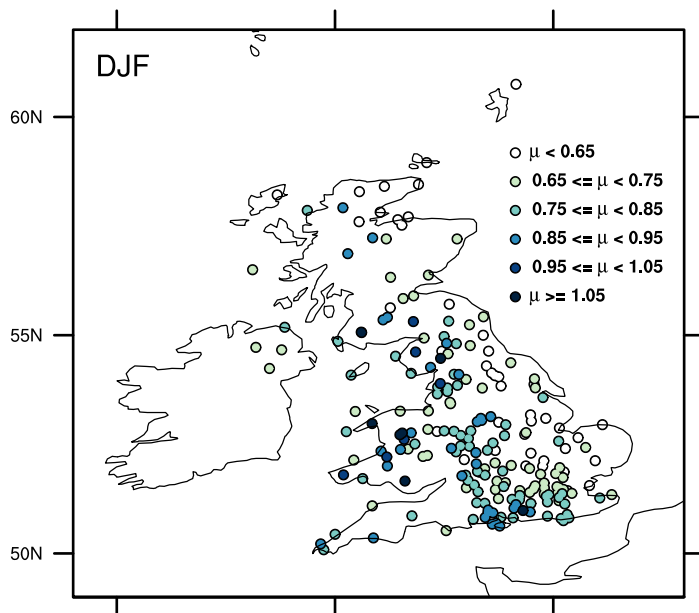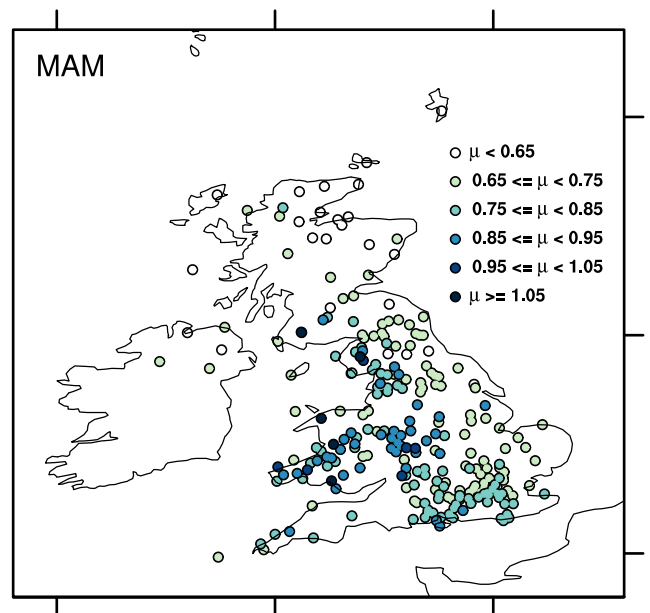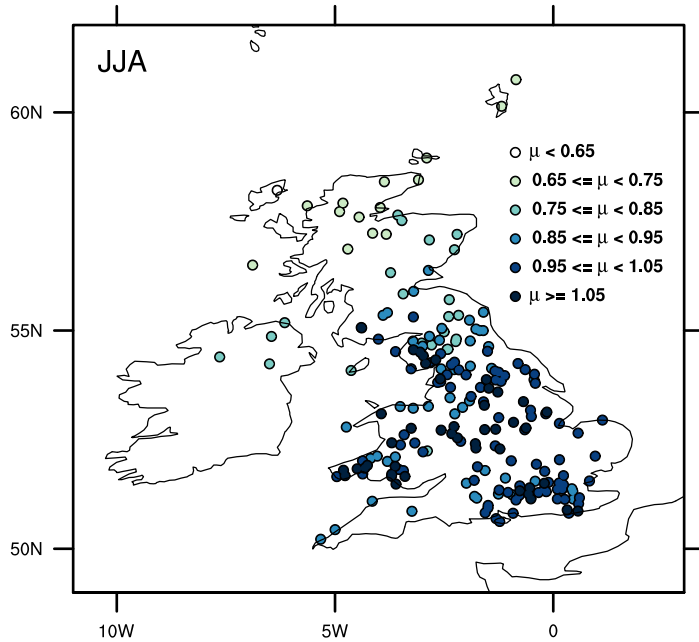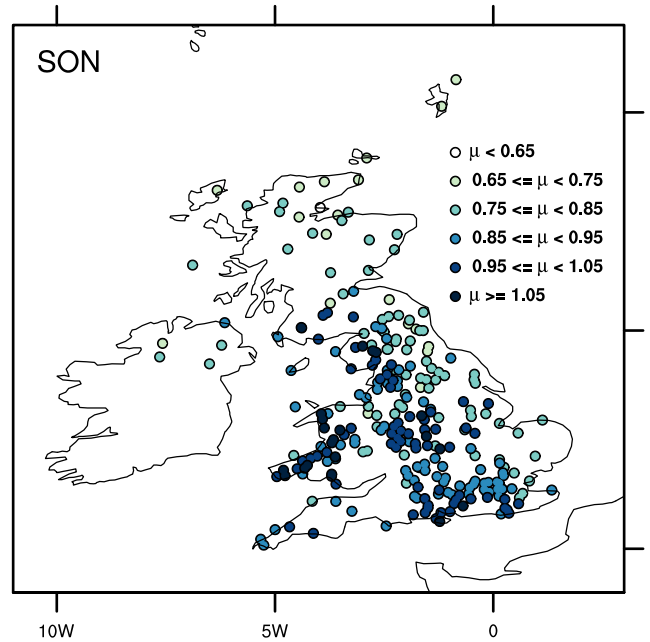

Supplement: Supplementary file 6 — Figure S5. Seasonal mean wet hour intensity. [file JOC-37-722-s006.pdf]

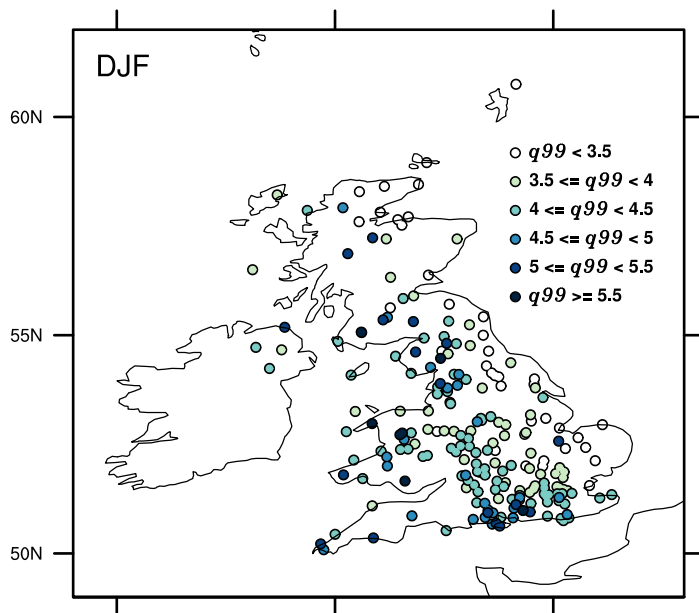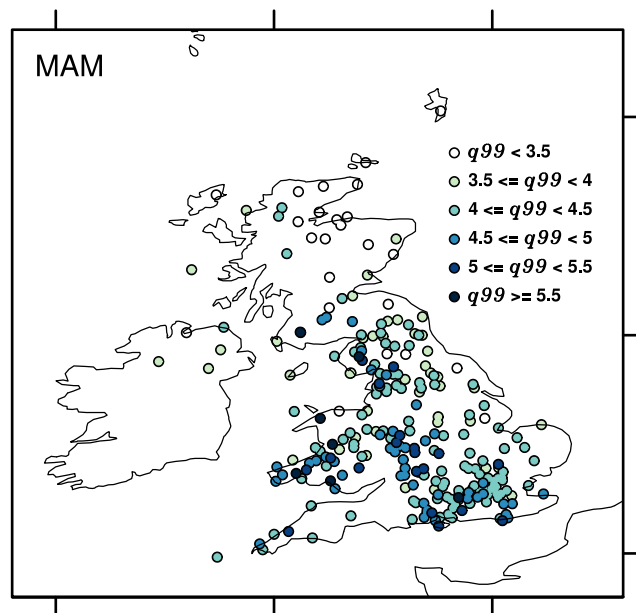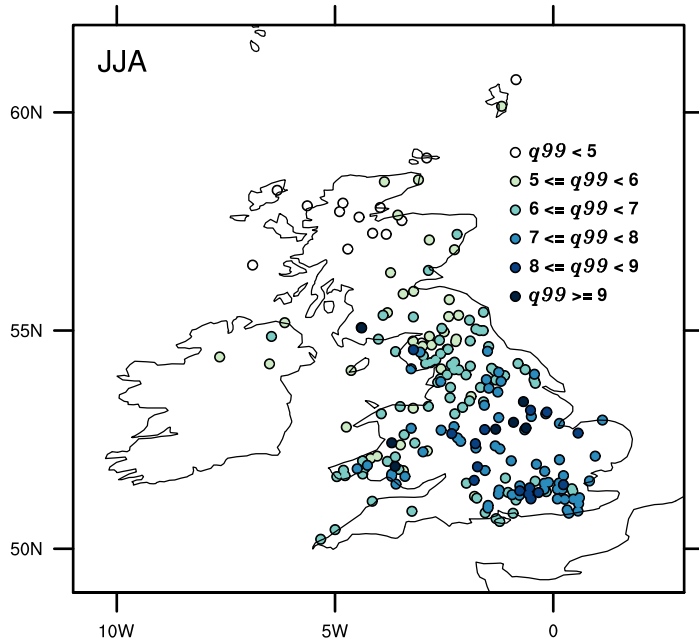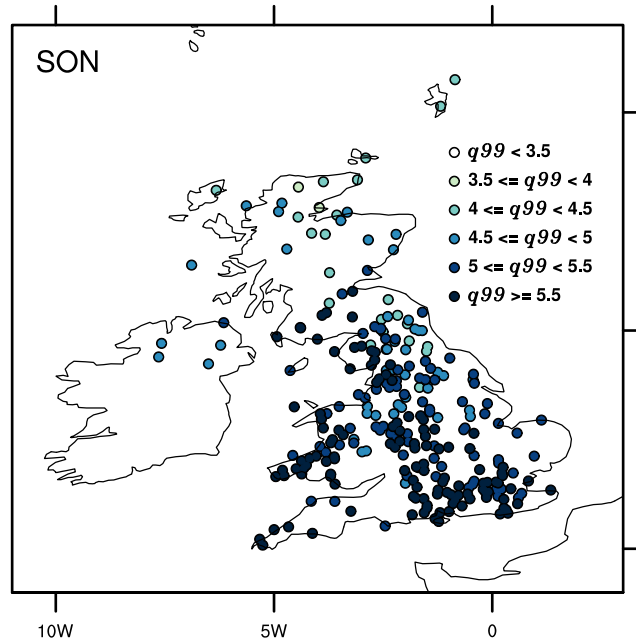

Supplement: Supplementary file 7 — Figure S6. Seasonal 99th percentile wet hour amount. [file JOC-37-722-s007.pdf]

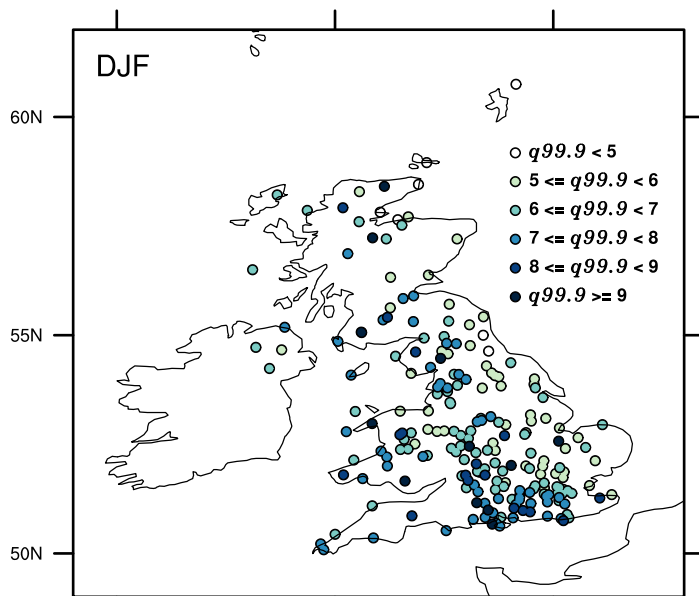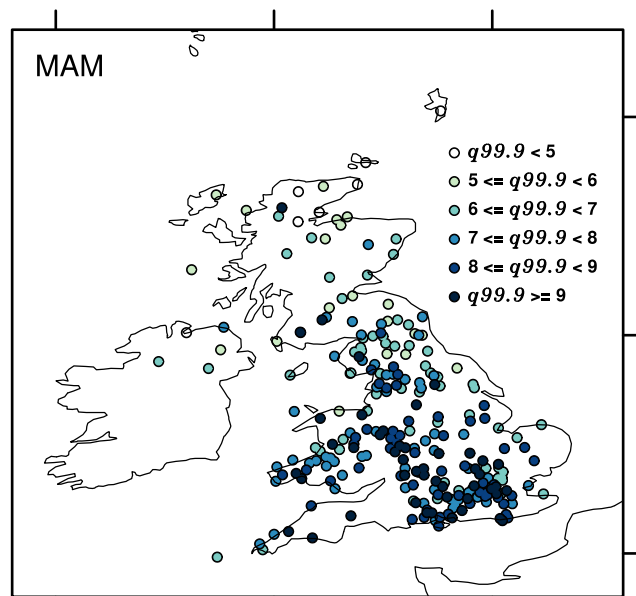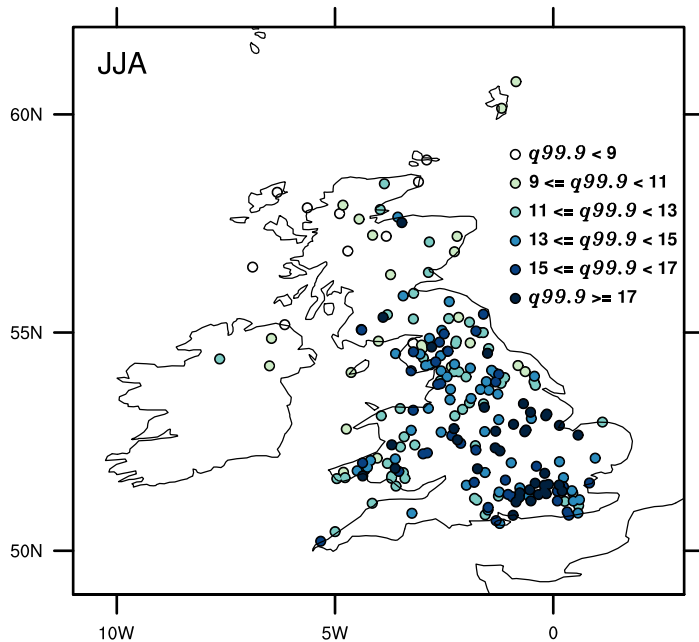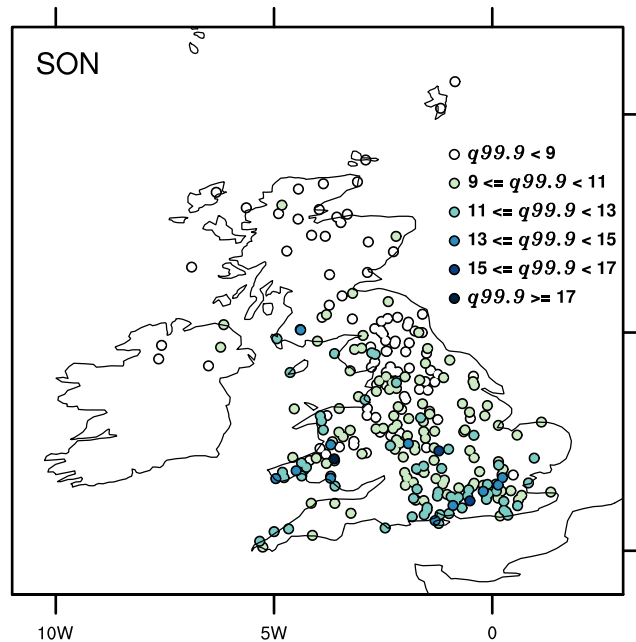

Supplement: Supplementary file 8 — Figure S7. Seasonal 99.9th percentile wet hour amount. [file JOC-37-722-s008.pdf]

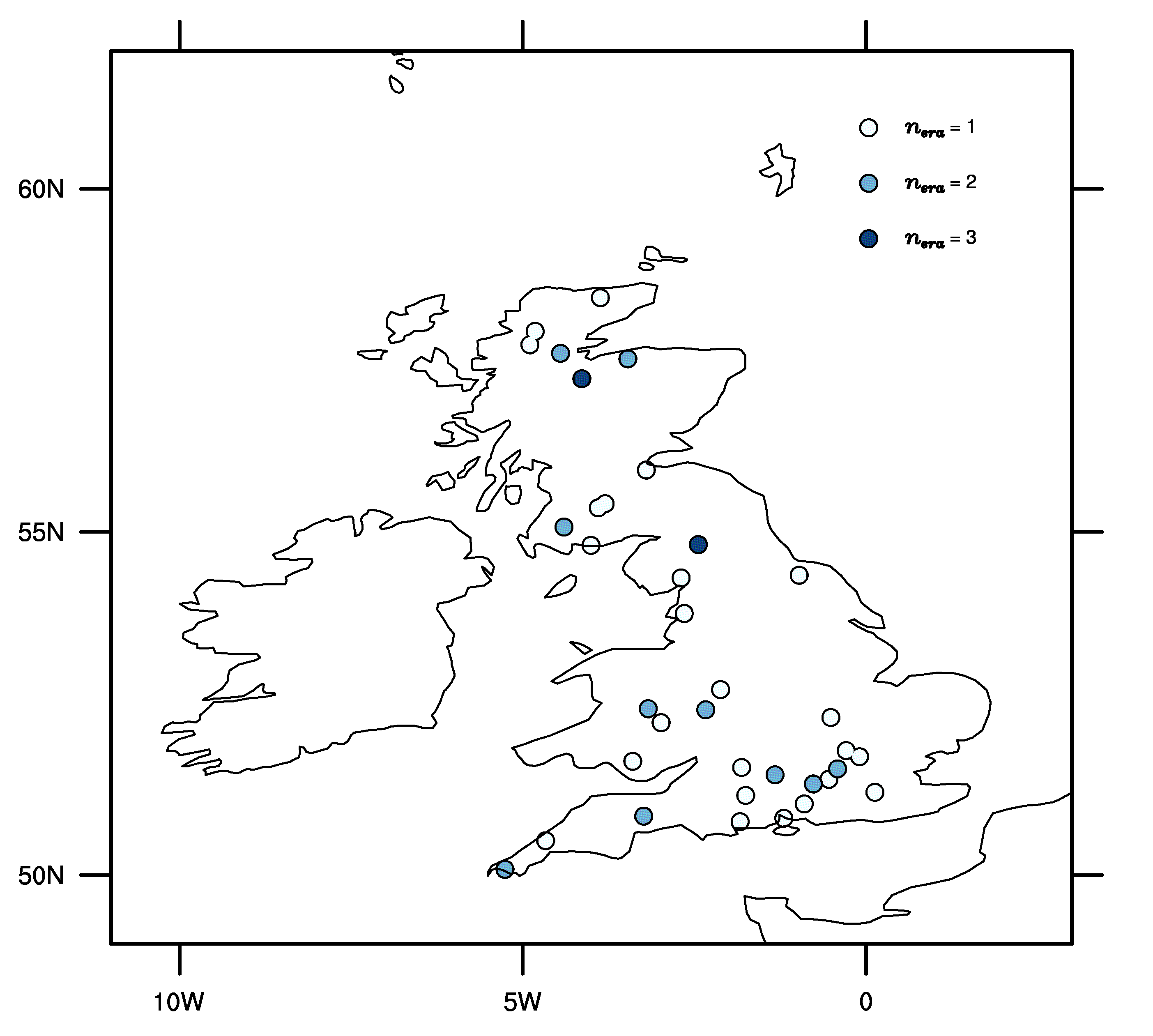

Supplement: Supplementary file 9 — Figure S8. Frequency of 1 h extreme rainfall alert (ERA) threshold events (≥30 mm h−1) after additional quality‐control procedures. [file JOC-37-722-s009.tif]

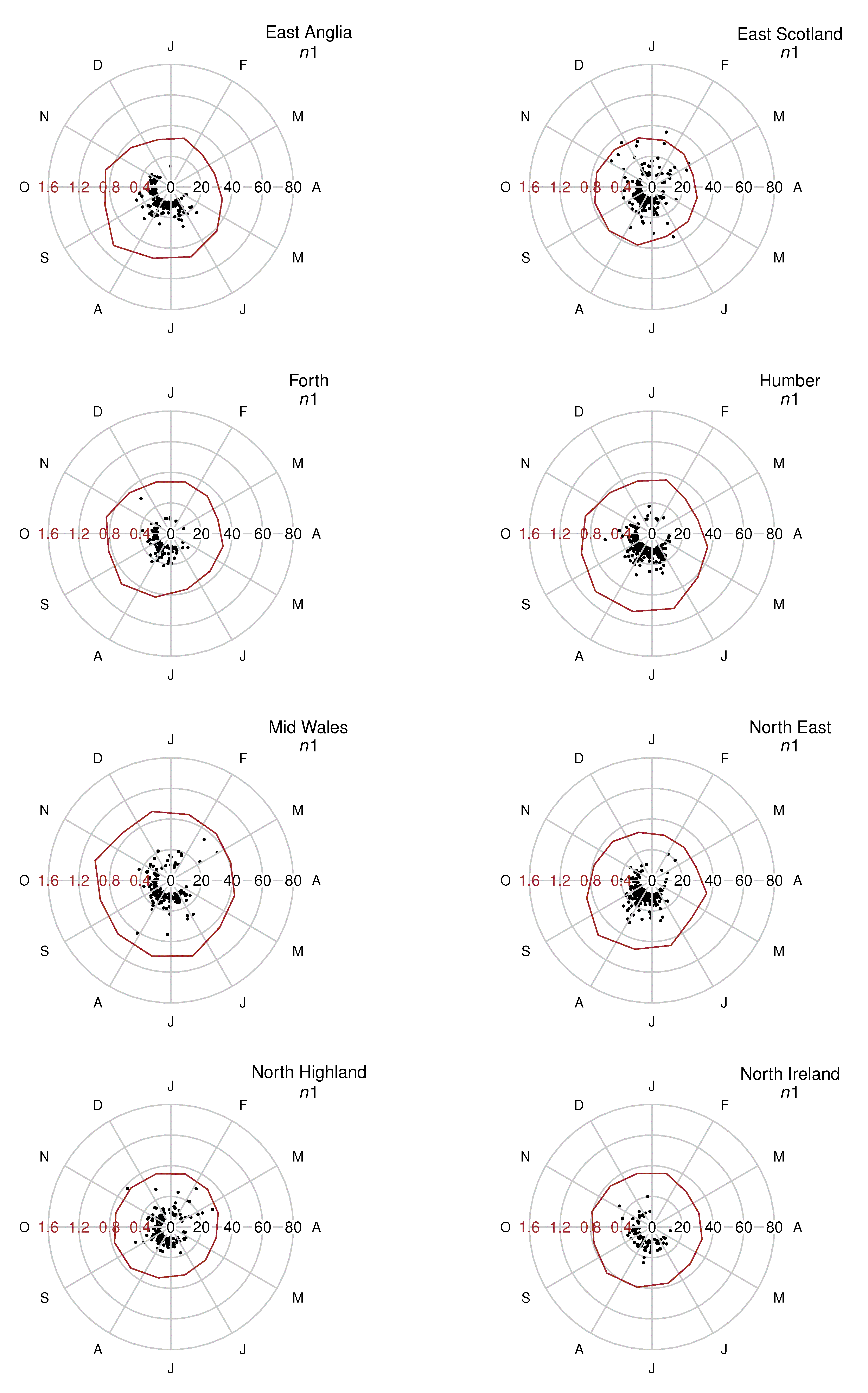

Supplement: Supplementary file 10 — Figure S9. Timing and magnitude of n1 events by extreme rainfall region. [file JOC-37-722-s010.zip › FigureS9/FigureS9a.tif]

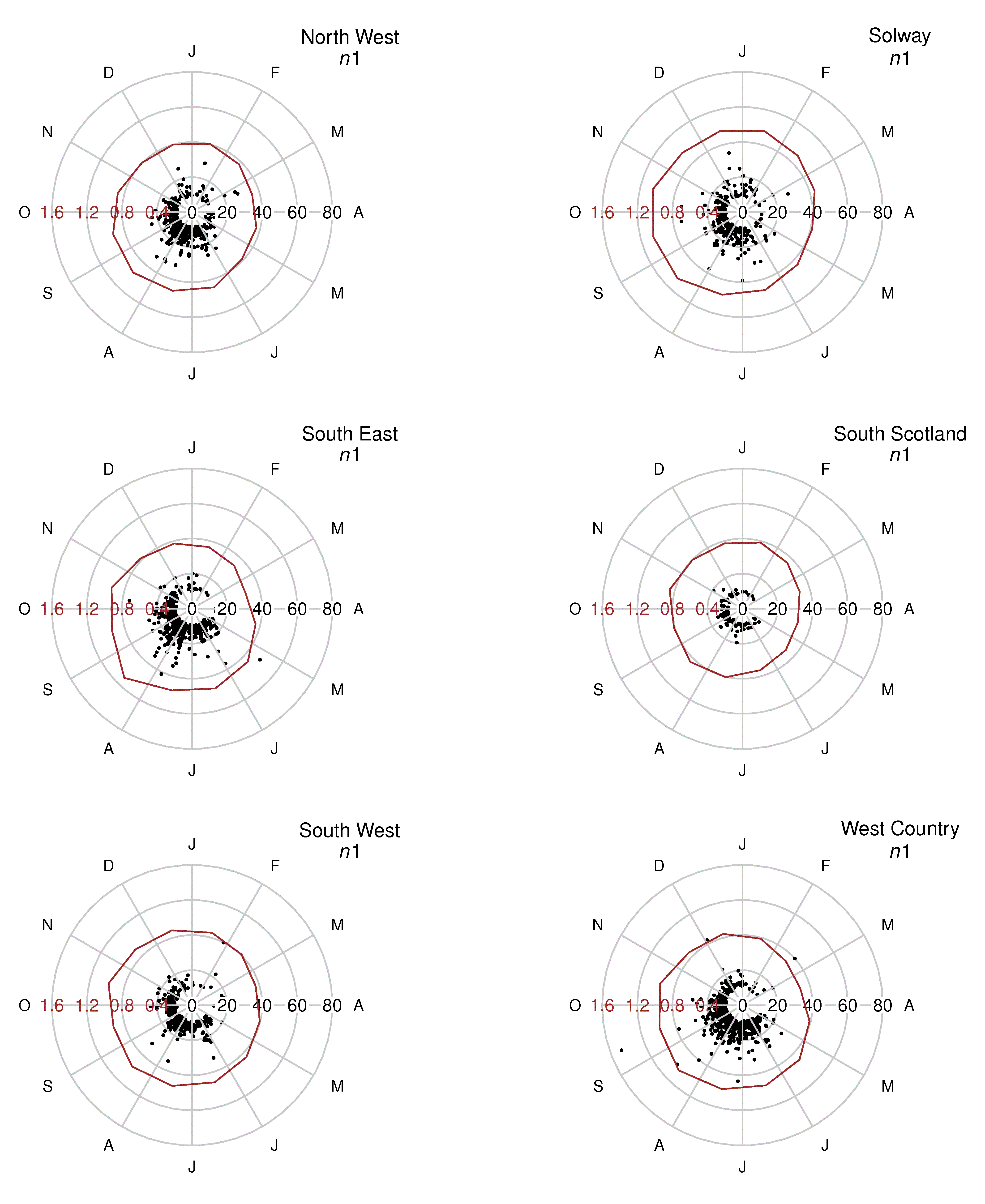

Supplement: Supplementary file 10 — Figure S9. Timing and magnitude of n1 events by extreme rainfall region. [file JOC-37-722-s010.zip › FigureS9/FigureS9b.tif]
